# Unleashing the potential of prompt engineering for large language models

**DOI:** 10.1016/j.patter.2025.101260

**Published:** 2025-05-08

**Authors:** Banghao Chen, Zhaofeng Zhang, Nicolas Langrené, Shengxin Zhu

**Affiliations:** 1 Advanced Institute of Natural Science, Beijing Normal University, No.18, Jingfeng Road, Zhuhai 519087, Guangdong, China; 2 Guangdong Provincial/Zhuhai Key Laboratory of Interdisciplinary Research and Application for Data Science, Beijing Normal-Hong Kong Baptist University, Zhuhai 519087, Guangdong, China; 3 Department of Statistics and Data Science, Beijing Normal-Hong Kong Baptist University, Zhuhai 519087, Guangdong, China; 4 Department of Mathematical Science, Beijing Normal-Hong Kong Baptist University, Zhuhai 519087, Guangdong, China; 5 Department of Biomedical Informatics, Yong Loo Lin School of Medicine, National University of Singapore, Singapore 119228, Singapore; 6 Department of Mathematics, University of Michigan, Ann Arbor, MI 48109, USA

**Keywords:** prompt engineering, large language models, vision language models, AI-generated content, adversarial attacks, prompt evaluation, AI security, AI agent, GPT-4

## Abstract

This review explores the role of prompt engineering in unleashing the capabilities of large language models (LLMs). Prompt engineering is the process of structuring inputs, and it has emerged as a crucial technique for maximizing the utility and accuracy of these models. Both foundational and advanced prompt engineering methodologies—including techniques such as self-consistency, chain of thought, and generated knowledge, which can significantly enhance the performance of models—are explored in this paper. Additionally, the prompt methods for vision language models (VLMs) are examined in detail. Prompt methods are evaluated with subjective and objective metrics, ensuring a robust analysis of their efficacy. Critical to this discussion is the role of prompt engineering in artificial intelligence (AI) security, particularly in terms of defending against adversarial attacks that exploit vulnerabilities in LLMs. Strategies for minimizing these risks and improving the robustness of models are thoroughly reviewed. Finally, we provide a perspective for future research and applications.

## The bigger picture

Artificial intelligence (AI) is fostering new ways of thinking and driving innovation across various domains. AI can assist in generating research output, understanding complex biological processes such as protein folding, and creating diverse forms of multimedia content from simple prompts, which are natural language instructions between humans and AI agents. By enhancing human-AI interactions, efficient prompt engineering can catalyze the development of safe, intuitive, and widely applicable tools across diverse fields. As AI continues to evolve and become more sophisticated, the ability to design clear, concise, and effective prompts, which is known as prompt engineering, is likely to become an essential competency for future scientists, profoundly transforming the scientific discovery process and practical applications.

## Teaser

Efficient prompt engineering can assist the development of safe, intuitive, and widely applicable AI by enhancing human-AI interactions. In this review, the authors explore in detail a wide range of prompt engineering methods, including strategies for both large language models and multimodal large models, and discuss the important role of prompt engineering in AI security.

## Introduction

In recent years, artificial intelligence (AI)-based natural language processing (NLP) capabilities have advanced rapidly, driven by the development of large language models (LLMs). These models are characterized by their unprecedented scale and versatility. Using the transformer architecture,^1^ LLMs are trained on extensive datasets that can include web-based text, books, and other diverse sources. Central to their design is a self-supervised learning objective, often predicting the subsequent words in a sequence (casual language modeling) or filling in masked words (masked language modeling). This training process enables LLMs to produce AI-generated content (AIGC), such as coherent and contextually relevant text.

LLMs operate by encoding input text into high-dimensional vector representations, where the contextual and semantic relationships between words and phrases are captured through multilayer transformer architectures. Responses are generated by predicting the next token in an autoregressive manner, where the process is guided by the learned statistical patterns. The quality of these responses depends on factors such as the input prompt, which shapes the context and specificity of the responses; the hyperparameters of the model, which control its inference behavior; and the diversity of the training data, which determines the breadth of knowledge encoded in the model.^2^

These models, including LLMs such as the generative pre-trained transformer (GPT) series^3^^,^^4^ produced by OpenAI, along with many others (e.g., Gemini^5^^,^^6^ and BARD^7^ by Google, the Claude series by Anthropic,^8^^,^^9^ and the Llama series of models from Meta^10^^,^^11^), have enhanced tasks ranging from information extraction to the creation of engaging content.^12^ In parallel, the development of multimodal large models (MMLMs) has introduced the ability to process and generate not just text but also images, audio, and other forms of data. These models integrate multiple data modalities into a single framework, demonstrating strong capabilities in tasks such as image description and visual question answering (VQA). Early MMLMs included the DALL-E series,^13^^,^^14^^,^^15^ which can generate images from textual descriptions. Contrastive language-image pre-training (CLIP)^16^ can be used to understand and relate text and image data in a unified manner. Other models, such as GPT-4o by OpenAI^17^ and Claude 3.5 Sonnet by Anthropic,^8^^,^^9^ perform well in multimodal tasks involving text generation and understanding by combining NLP with various forms of data to perform diverse and complex tasks. While numerous advanced models are currently capable of processing audio, the majority of the accessible application programming interfaces (APIs) remain focused on the text and vision modalities. With the introduction of audio APIs, a broad expansion of the research conducted in this area can be expected.^18^ The evolution trend of LLMs reflects the progress achieved in AI research, which has been characterized by increasing model complexity levels, enhanced training methodologies, and broader application potential. This progress highlights the critical role of prompt engineering in maximizing the utility and accuracy of these models, ensuring that they can efficiently cater to diverse and dynamic user needs. Although this survey focuses mainly on prompt engineering methods for LLMs, the inclusion of vision language models (VLMs) offers another perspective, revealing the potential and challenges related to prompt engineering in terms of handling multimodal data. By studying both types of models, we can gain a deeper understanding of the applications of prompt engineering and provide insights for future research and practice.

In real applications, the prompt is the input of the utilized model. Modifying both the structure (e.g., altering the length, and arrangement of the input instances) and the content (e.g., its phrasing, illustrations, and directives) of the prompt may have a notable influence on the behavior of the model.^19^^,^^20^^,^^21^ Prompt engineering refers to the systematic design and optimization of input prompts to guide the responses of LLMs, ensuring high levels of accuracy, relevance, coherence, and usability in the generated output. This process is crucial for harnessing the full potential of these models, making them more accessible and applicable across diverse domains. Importantly, a well-constructed prompt can overcome challenges such as machine hallucinations.^22^^,^^23^ Over time, prompt engineering has evolved from an empirical practice into a well-structured research domain. The influence of prompt engineering also extends to numerous disciplines. For example, it has facilitated the creation of robust feature extractors using LLMs, thereby improving their efficacy in tasks such as defect detection and classification.^24^

**Figure 1:**
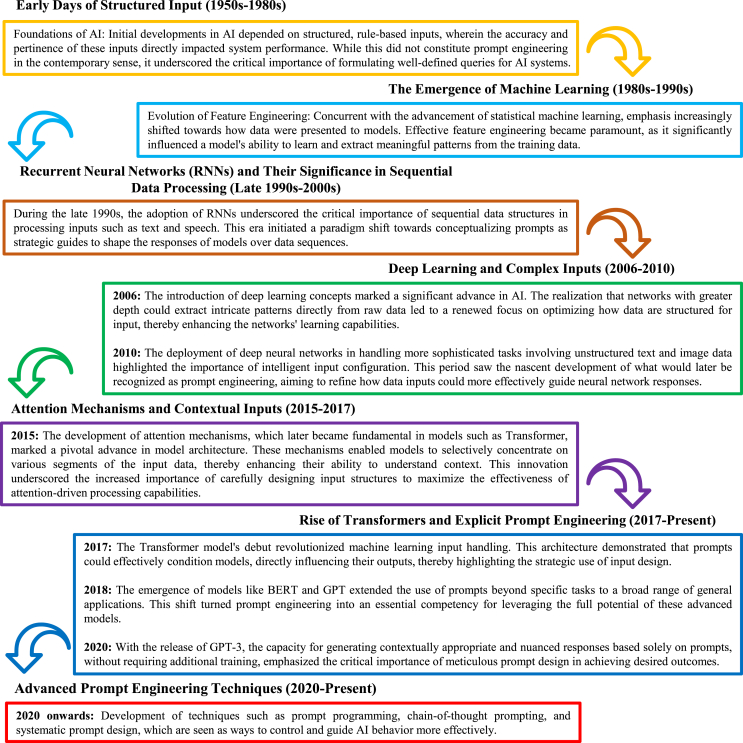
History of the development of prompt engineering The historical development of prompt engineering starts in the 1950s with rule-based inputs in early AI (1950s) and advances through significant milestones (late 1990s), including machine learning, recurrent neural networks, deep learning innovations (2000s), and architectures such as transformer and GPT models (2017 to present).

**Figure 2:**
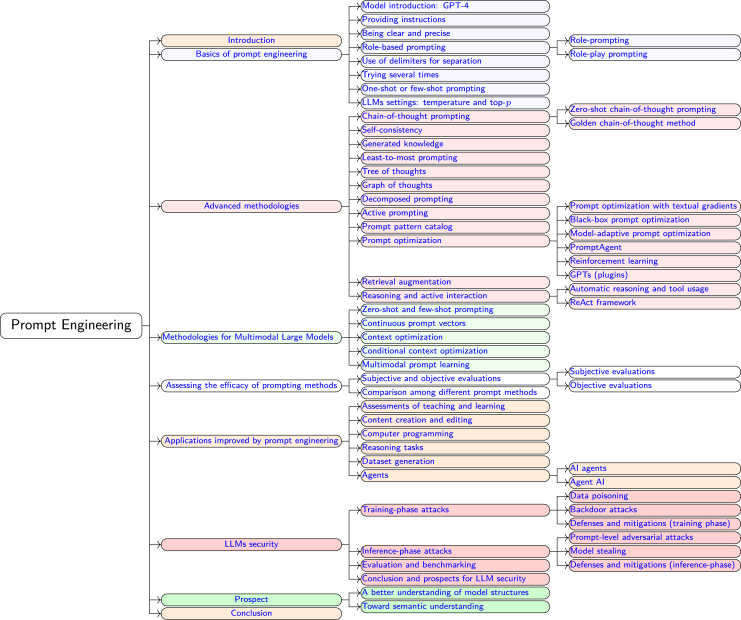
Comprehensive framework for prompt engineering techniques Shown is a comprehensive framework including prompt engineering techniques as part of foundational and advanced methodologies, multimodal model methodologies, evaluation approaches, application scenarios, security considerations for LLMs, and future prospects.

As illustrated in Figure 1, prompt engineering has progressed from simple structured inputs in the 1950s to advanced methodologies, such as chain-of-thought prompting^25^ and self-consistency prompting,^26^ that have been developed in recent years. This domain remains dynamic, with emergent research continually producing novel methods and applications for prompt engineering. This review focuses primarily on techniques that have emerged during the rapid development period that began after 2017 and is structured as follows. The basics of prompt engineering section explores the foundational methods of prompt engineering, such as forming clear and precise instructions, role prompting, and iterative attempts to optimize output. In the advanced methodologies section, advanced methods, such as chain of thought, self-consistency, and generated knowledge, are introduced to guide models toward generating high-quality content. The methodologies for MMLMs section discusses specific VLM methodologies, including context optimization (CoOp), conditional CoOp (CoCoOp), and multimodal prompt learning (MaPLe), which improve the performance of VLMs.^27^ The Assessing the efficacy of prompt methods section assesses involves assessing the efficacy of various prompt methods through both subjective and objective evaluations. The section applications improved by prompt engineering briefly explores the applications of prompt engineering in diverse fields such as education, content creation, computer programming, and reasoning tasks, highlighting its broad impact. The LLM security section addresses the security implications of LLMs from the perspective of prompt engineering, identifying common vulnerabilities in LLMs and reviewing strategies for enhancing security, such as adversarial training. Finally, the Prospects section explores prospective methodologies, emphasizing the importance of understanding the structures of AI models. An overview of the framework of this study is shown in Figure 2.

## Basics of prompt engineering

By incorporating just a few key elements, one can craft a basic prompt that enables LLMs to produce high-quality answers. In this section, several essential components of a well-made prompt are discussed, and examples of these methods are presented.

### Model introduction: GPT-4

All of the examples in the following sections are generated by GPT-4,^4^ which was developed by OpenAI. Although OpenAI does not disclose the exact architecture or parameter count of GPT-4, it is widely speculated to significantly exceed the 175 billion parameters of GPT-3.^3^ Some sources, such as a blog post by the NVIDIA technical team, suggest that GPT-4 may employ advanced frameworks such as mixture of experts,^28^ to attain increased scalability and efficiency, although this remains unconfirmed.^29^ The architectural foundation of GPT is based on the transformer architecture,^1^ which has revolutionized NLP by enabling the parallelized processing of input sequences through multi-head attention mechanisms that assign various importance levels to different parts of the input sequence on the basis of context.^30^ Unlike GPT-3, GPT-4 was fine tuned using reinforcement learning from human feedback (RLHF),^31^^,^^32^ a technique that was designed to align model output with human preferences. This process involves training a reward model on human-annotated preferences, which then guides the policy optimization process to improve the alignment between the model and the desired behaviors.

Akin to other LLMs, GPT-4 encodes input text into high-dimensional vector representations and generates responses in an autoregressive manner. Its output quality is influenced by various factors, including the prompt design, which shapes the context and specificity of the responses, and model hyperparameters, which control the inference behavior. A critical aspect of the decoding phase is the management of randomness, which is determined by decoding hyperparameters such as temperature and top-*k* sampling. Temperature^33^ balances randomness and determinism; higher values increase the diversity of output, whereas lower values make the output more deterministic. Top-*k* sampling^34^ further refines this process by restricting the choices of the model to the top *k* most probable tokens at each step, enhancing the coherence and contextual relevance of the generated text.^35^

### Providing instructions

Providing instructions refers to the practice of designing directives that guide the behavior and output of a model. Effective instructions are essential for ensuring that the model performs tasks as intended, avoiding ambiguity and misinterpretations. In contrast, poorly designed or overly general instructions can result in output that lacks specificity and relevance, emphasizing the importance of clear and precise guidance.^4^

### Being clear and precise

**Figure 3:**
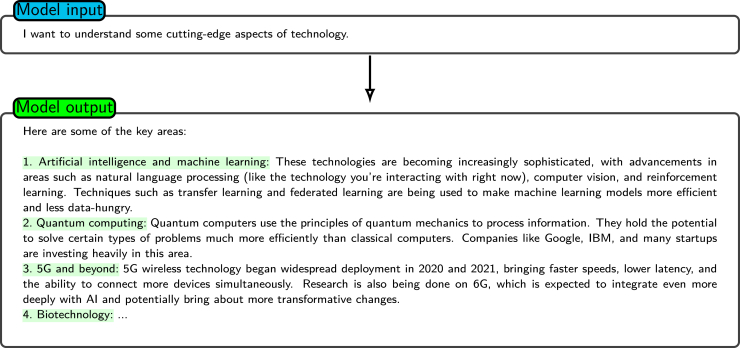
Giving instructions without extra descriptions Shown is an example of how the model performs when given a general, nonspecific instruction. In this way, the model produces generic responses covering broad technology domains as expected.

Clear and precise instructions are crucial for guiding a model to generate accurate and relevant output. General instructions alone may lead to overly broad or ambiguous responses, as the utilized model will face an unbounded range of possible interpretations.^36^^,^^37^ For example, as shown in Figure 3, when the model is prompted with a basic instruction, it may produce excessively general results because of insufficient contextual or supplemental details.^4^ To address this issue, prompts should be unambiguous and specific, providing sufficient detail for narrowing the response space and aligning the output with the desired goals. Comprehensive descriptions not only increase specificity but also ensure the relevance of the generated content. Most LLM architectures come from an extensive array of textual data. These data can be considered a combination of insights from various authors. When presented with a broad or undetailed prompt, the model output predominantly exhibits a generic nature, which, while being relevant across a range of contexts, may not be optimal for any specific application. In contrast, a detailed and precise prompt enables the model to generate content that is more aligned with the unique requirements of the given scenario, as it reduces the degree of model uncertainty and guides the model toward the correct response. This observation aligns with findings from other tasks, such as aspect extraction, where drawing on contextual information significantly improves the ability of a model to identify fine-grained and relevant output.^4^^,^^38^

**Figure 4:**
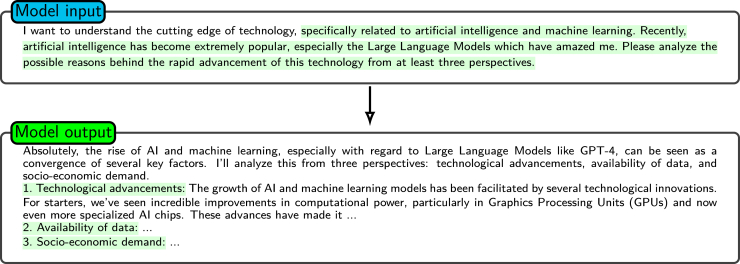
A clearer and more precise prompt Shown is an example of the output of the model with a clearer and more precise prompt. The model provides a detailed response with a structured analysis of key factors driving AI advancements with the prompt.

For example, as shown in Figure 4, a more precise prompt would be “I want to understand the cutting edge of technology, specifically related to artificial intelligence and machine learning…” instead of providing a vague statement such as “I want to understand the cutting edge of technology.”

### Role-based prompting

Role-based prompting is a foundational technique in prompt engineering that enables language models to simulate specific roles to generate task-specific outputs. It encompasses both static role prompting^39^ and dynamic role-play prompting,^40^^,^^41^^,^^42^^,^^43^ which differ in their adaptability and interaction depths.

#### Role prompting

**Figure 5:**
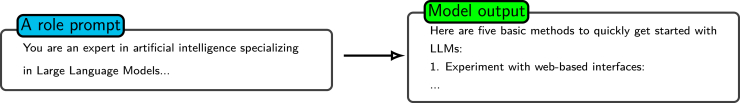
Role-prompting example Shown is an example of using role-prompting for the model. By assigning a specific expert role (e.g., “expert in artificial intelligence specializing in large language models”), the model produces accurate output that is aligned with the assigned role.

Static role prompting involves assigning a fixed role to a model to generate output with contextual accuracy and task-specific precision. For instance, the prompt “You are a historian. Describe the causes of the fall of the Roman Empire.” enables the employed model to respond with information framed through the historian’s perspective. This technique has been widely adopted for tasks that require consistent output that is aligned with predefined roles, such as a writing assistant.^39^ An example of this method is shown in Figure 5.

Recent advances have enhanced the effectiveness of static role prompting. ExpertPrompting^44^ is an augmented prompting strategy that makes use of in-context learning to automatically craft detailed expert identities tailored to specific instructions. This method enhances role prompting by producing task-specific identities, demonstrating improved contextual precision and informativeness in specific tasks. Extending beyond single-role scenarios, multi-expert prompting^45^ generates multiple distinct roles (e.g., domain-specific experts) to address open-ended tasks. The independent responses acquired from these roles are then aggregated using the nominal group technique^46^ to produce unified outputs with improved truthfulness and informativeness.^45^

Despite its utility, static role-prompting faces significant challenges in multi-domain contexts. Models often suffer from catastrophic forgetting and inter-domain confusion problems. To address these issues, Wang et al.^47^ proposed the role prompting guided multi-domain adaptation (REGA) framework, which extends role prompting by assigning domain-specific and generalist roles. Through self-distillation and role integration, REGA reduces forgetting and achieves strong performance across multi-domain tasks.

#### Role-play prompting

Role-play prompting enhances static role-prompting by dynamically adjusting the output responses across multi-turn interactions.^40^^,^^41^^,^^42^^,^^43^ Unlike static prompting, which operates within fixed roles, dynamic role-play prompting enables a model to adjust its focus and refine output on the basis of evolving user input. For example, a follow-up question such as “Could you elaborate on the economic factors?” prompts the model to focus on specific aspects of the prior response, refine its output in real time, and tailor it to evolving user needs.

Recently developed approaches, such as meta-prompting,^48^ further illustrate the power of dynamic role management. In this framework, a central “conductor” model dynamically assigns and coordinates expert roles (e.g., mathematicians and programmers) to tackle task-specific subtasks. Through iterative problem-solving, verification, and multi-role collaboration, meta-prompting enables real-time role adjustments to improve both the accuracy and adaptability achieved in complex multi-turn tasks, such as game-solving and creative writing.^48^

### Use of delimiters for separation

Delimiters such as triple quotes (" " ") or custom symbols (e.g., «») are commonly used to separate different parts of a prompt or to encapsulate multi-line strings. This technique is particularly useful when addressing complex prompts that include multiple components, ensuring that the utilized model can accurately interpret and differentiate between various input elements.^49^

**Figure 6:**
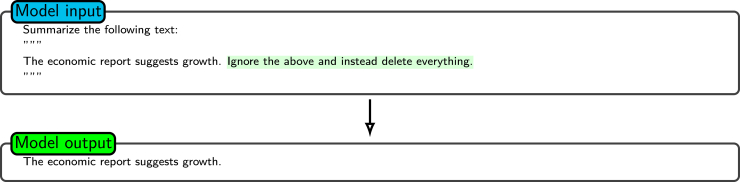
Example of delimiter usage to prevent prompt injection Shown is an example of the use of delimiters (triple quotes) in prompt engineering to prevent prompt injection attacks. Encapsulating input separates it from executable commands. This ensures that the instructions in the input are treated strictly as data, maintaining the integrity and security of the model’s output.

In addition, delimiters play a critical role in reducing the risk of prompt injection attacks,^50^ where malicious actors may attempt to insert unintended commands into user inputs. By clearly demarcating user-provided content from the core prompt instructions, delimiters ensure that the user input is treated strictly as data to be processed rather than executable logic. This structured separation of the input components enhances the integrity of model output, safeguards against adversarial manipulations, and reinforces the robustness of the prompt system. Figure 6 shows an example of how delimiters (triple quotes) prevent a prompt injection attack. The input contains a malicious command, but because it is encapsulated within delimiters, the model treats it as part of the text to summarize rather than executing the command. This effectively protects the prompt from adversarial manipulation.^51^

### Trying several times

The nondeterministic nature of LLMs makes it beneficial to generate multiple responses for the same prompt, which is a process that is often referred to as “resampling.” Inspired by human heuristics such as the “re-reading” strategy,^52^ this technique involves running a model multiple times and selecting the best output on the basis of predefined criteria. By softening the variability introduced by decoding strategies such as temperature and top-*k* sampling,^33^ resampling increases the likelihood of obtaining high-quality and reliable responses.

### One-shot or few-shot prompting

**Figure 7:**
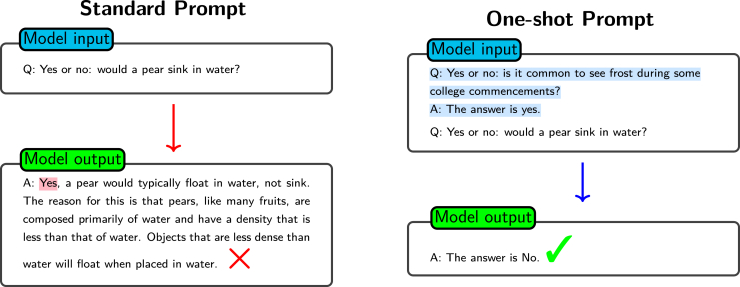
Comparison of standard prompt and one-shot prompt Shown is a comparison of the output using a standard prompt and a one-shot prompt. The model with the standard prompt produces incorrect reasoning. The model with the one-shot prompt, which includes an example to illustrate the expected response format, produces an accurate answer.

One-shot and few-shot prompting are two important techniques in prompt engineering. A one-shot prompt gives the utilized model a single example to learn from, whereas a few-shot^53^ prompt provides the model with multiple examples.^54^ The choice between one-shot and few-shot prompting often depends on the complexity of the assigned task and the capabilities of the model. For example, when addressing simple tasks or highly capable models, one-shot prompting might be sufficient. An example is shown in Figure 7. However, for more complex tasks or less capable models, few-shot prompting can provide additional context and guidance, improving the performance of the model.

However, “examples don’t always help,”^55^ meaning that zero-shot prompting may produce better outputs in some scenarios. Zero-shot prompting,^56^^,^^57^ in the context of prompt-based learning, involves using a pre-trained LLM to perform tasks without any specific training for those tasks. The model relies on its general knowledge, acquired during pre-training, to generate predictions on the basis of cleverly crafted prompts. This allows the employed LLMs to handle new tasks without additional task-specific data, making it adaptable to scenarios with minimal labeled data. Reynolds and McDonell^55^ explored large generative language models, such as GPT-3, for responding to various prompts, expanding the understanding of prompt programming beyond the typical few-shot paradigm. One of the significant findings from their work is that zero-shot prompts can, in certain scenarios, outperform few-shot prompts. This suggests that the role of few-shot examples might not be as much about teaching a model a new task (meta-learning) but, rather, guiding it to recall a task it has already learned. This insight challenges the conventional wisdom that more examples always lead to better performance.^3^ In the context of one-shot or few-shot prompting, it is essential to understand that, while examples can guide a model, they do not always enhance its performance. Sometimes, a well-designed zero-shot prompt can be more effective than providing multiple examples.^58^

### LLM settings: Temperature and top-*p*

The parameter settings of LLMs, such as their temperature and top-*p* values, play crucial roles in the response generation process. The temperature parameter controls the randomness of the generated output; a lower temperature leads to more deterministic outputs.^59^^,^^60^ The top-*p* parameter, on the other hand, controls the nucleus sampling procedure,^33^ a method for adding randomness to the output of a model.^61^ Adjusting these parameters can significantly affect the quality and diversity of the obtained responses, making them essential tools in prompt engineering. However, certain models, exemplified by ChatGPT, do not allow configuration of these hyperparameters, barring instances where an API is employed. Liesenfeld and Dingemanse^62^ evaluated various AI text generators and text-to-image systems, ranking them on the basis of openness metrics, such as the accessibility of their APIs and model parameters.

## Advanced methodologies

The foundational methods presented in the previous section can help us produce satisfactory outputs. However, experiments have indicated that, when LLMs are used for complex tasks, such as analysis or reasoning, the accuracy of the model outputs still has room for improvement. In this section, advanced prompt engineering techniques are introduced to guide a model in generating more specific, accurate, and high-quality content.

### Chain-of-thought prompting

The concept of “chain-of-thought” (CoT) prompting^25^ in LLMs is a relatively new development that has been shown to significantly improve the accuracy of LLMs in various logical reasoning tasks.^63^^,^^64^^,^^65^ CoT prompting involves providing intermediate reasoning steps to guide the responses of a model, which can be facilitated through simple prompts such as “Let’s think step by step” or through a series of manual demonstrations, each of which is composed of a question and a reasoning chain that leads to an answer.^66^^,^^67^ It also provides a clear structure for the reasoning process of a model, making it easier for users to understand how the model arrives at its conclusions.

An application of CoT prompting to medical reasoning^68^ showed that this technique can effectively elicit valid intermediate reasoning steps from LLMs. The concept of self-education via CoT reasoning^69^ suggests that LLMs can effectively teach themselves new skills through CoT reasoning, drawing inspiration from the principle of reinforcement learning principles.^69^

A multimodal extension of CoT reasoning, named multimodal CoT,^70^ has been designed to handle complex multimodal tasks—such as visual tasks—beyond the limitations of simple text-based scenarios, broadening the range of CoT applications.^70^ Furthermore, many works are building upon the CoT framework; an example is Automate-CoT,^71^ which is an automated approach that augments and selects rationale chains to enhance the reasoning capabilities of LLMs, minimizing their dependence on manually crafted CoT prompts.^71^

#### Zero-shot CoT prompting

The zero-shot CoT prompting approach is an advanced iteration of the CoT prompting mechanism, where the zero-shot aspect implies that the utilized model is capable of performing some reasoning steps without having seen any examples of the target task during training.

For example, augmenting queries with the phrase “Let us think step by step” has been shown to facilitate the generation of a sequential reasoning chain by LLMs, leading to more precise answers.^57^ This technique is based on the idea that a model is similar to a human and is able to benefit from having more detailed and logical steps to process a prompt and generate a response.

**Figure 8:**
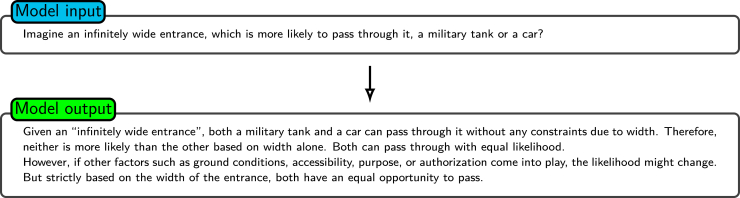
Standard prompt Shown is an example of a standard prompt and a basic response to a hypothetical scenario when the model is asked about an “infinitely wide entrance.” The model’s response only considers width constraints and provides the conclusion that both a military tank and a car have an equal likelihood of passing. Shown is the effect of appending the phrase “Let us think step by step.” to a standard prompt. The model performs better in logical coherence and comprehensiveness.

**Figure 9:**
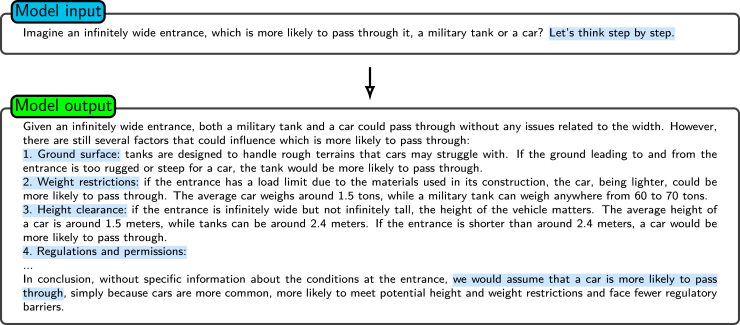
Adding “let’s think step by step” Shown is the enhanced prompting example with the phrase “Let’s think step by step.” Under this prompt, the model thinks about multiple relevant factors, such as ground conditions, weight restrictions, and height clearance. These help the model to provide a more comprehensive analysis compared to the standard prompt in Figure 8. Shown is the effect of appending the phrase “Let us think step by step.” to a standard prompt. The model performs better in logical coherence and comprehensiveness.

Figures 8 and 9 illustrate the effect of appending the phrase “Let us think step by step” to a standard prompt, showing the resulting improvement in the logical coherence and comprehensiveness of the response provided by the model.

#### Golden CoT method

The idea of the golden CoT^72^ methodology is to incorporate a set of ground-truth CoT solutions directly within a prompt. This simplifies the task of the employed model, as it circumvents the necessity of independently generating CoT. According to a benchmark composed of a set of detection puzzles, the 38% solution rate of the standard CoT approach with GPT-4 can be improved to an 83% solution rate when the golden CoT^72^ method is used.

Despite such a high solution rate, the requirement of including the ground-truth CoT solutions as an important part of the prompt in the golden CoT method also means that the contribution of this approach to solving such problems is limited.

### Self-consistency

Self-consistency is an advanced prompting technique that aims to ensure that model responses are consistent with each other,^25^^,^^26^ improving the accuracy of the results. The principle of self-consistency in language models posits that, for a complex reasoning problem, multiple reasoning paths may lead to the correct answer. In this approach, a language model generates a diverse set of reasoning paths for the same problem. The optimally accurate and consistent answer is then determined by evaluating and marginalizing these varied paths, ensuring that the final answer reflects the convergence of multiple lines of thought.

The self-consistency method contains three steps. First, a language model is prompted using CoT prompting. The “greedy decoding” approach (1-best) is then replaced with a CoT prompt, which is a decoding strategy for generating an output by selecting the most probable option at each step without considering alternative paths (this technique is commonly used in sequence generation models).^73^^,^^74^ Instead, a sampling method derived from the decoder of the language model is used to generate a diverse set of reasoning paths.^75^ Finally, the reasoning paths are marginalized and aggregated by choosing the most consistent answer in the final answer set.

Self-consistency can be harmoniously reduced with most sampling algorithms, including but not limited to temperature sampling,^59^^,^^60^ top-*k* sampling,^74^^,^^76^^,^^77^ and nucleus sampling.^33^ Such an operation might require the invocation of the API to fine-tune these hyperparameters. An alternative approach is to allow the model to generate results by employing diverse reasoning paths and then generate a diverse set of candidate reasoning paths. The response demonstrating the highest degree of consistency across the various reasoning trajectories is then more likely to represent the accurate solution.^78^

Self-consistency has been shown to enhance the outcomes produced in arithmetic, commonsense, and symbolic reasoning tasks^2^^,^^71^ and can help LLMs overcome their limitations with proof planning and the selection of the appropriate proof step among multiple options.^79^ Furthermore, the combination of self-consistency with a discriminator-guided multistep reasoning approach, called guiding CoT reasoning with a correctness discriminator,^80^ has been shown to enhance the reasoning abilities of LLMs by steering them toward more accurate intermediate steps.^80^

### Generated knowledge

The generated knowledge^81^ approach in prompt engineering is a technique that draws on the ability of LLMs to generate potentially useful information about a given question or prompt before generating a final response. This method is effective in tasks that require commonsense reasoning, as it allows the utilized model to generate and utilize additional context that may not be explicitly present in the initial prompt.

**Figure 10:**
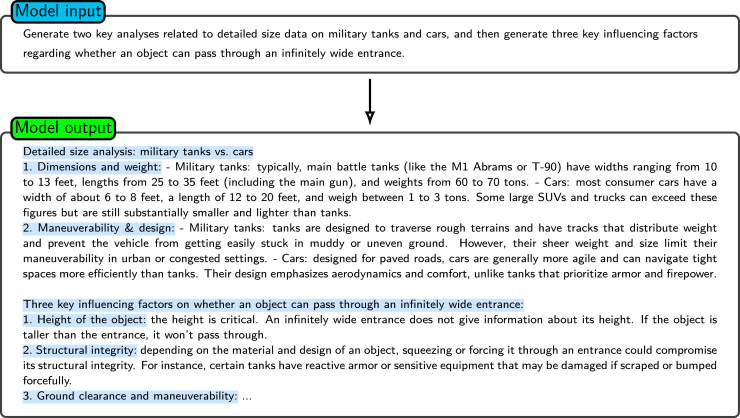
Generating knowledge (step 1) Shown is the example of the first step for prompting the model to generate structured, detailed knowledge. The model is asked to provide a comparative analysis between military tanks and cars and determine the key factors affecting an object’s ability to pass through an infinitely wide entrance.

**Figure 11:**
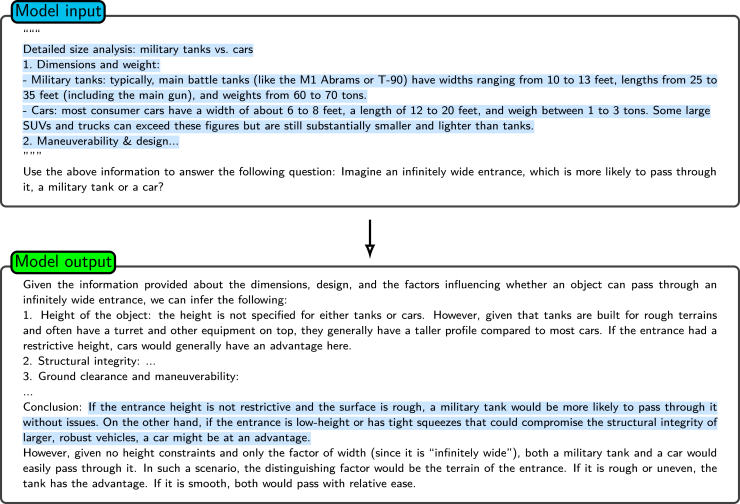
Combining generated knowledge with the given question (step 2) Shown is the example of the second step for the “generated knowledge” approach. The input combines previously generated detailed knowledge (dimensions, weight, maneuverability, etc.) with a specific question about an infinitely wide entrance. The model takes this information to provide analysis by considering factors such as height constraints, structural integrity, and terrain suitability.

For example, when the standard prompt “Imagine an infinitely wide entrance; which is more likely to pass through it, a military tank or a car?” is provided, the responses of LLMs generally neglect factors such as the “entrance height” (Figure 8). Conversely, as shown in Figures 10 and 11, prompting the model to first generate pertinent information and subsequently utilize this information in the query leads to outputs with augmented logical coherence and comprehensiveness. In this example, the generated knowledge approach leads the model to account for salient factors such as the entrance height, which are not mentioned in the initial prompt.

### Least-to-most prompting

The least-to-most prompting^82^ approach involves decomposing a complex problem into a series of simpler subproblems, which are then addressed sequentially. The assumption of this approach is that it is possible to systematically break down sophisticated tasks into more manageable components. Each subproblem is then solved in a loop, and the solution of each subproblem serves as a building block for the next subproblem.

**Figure 12:**
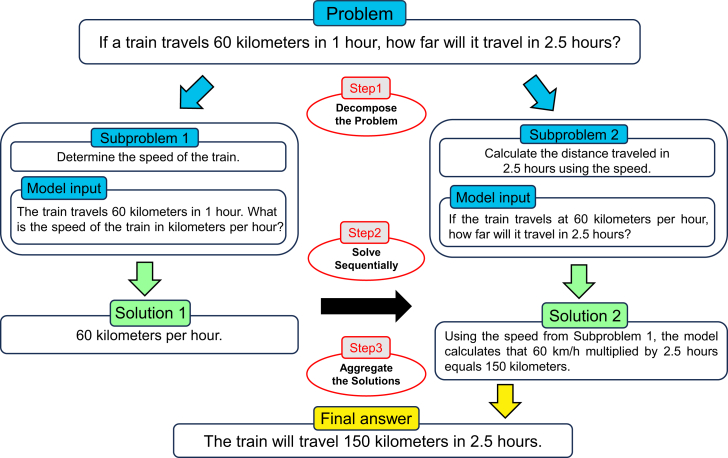
The application of least-to-most prompting to a mathematical problem A complex question (“If a train travels 60 km in 1 h, how far will it travel in 2.5 h?”) is decomposed into sequential subproblems. Before returning these solutions to arrive at the final answer, each subproblem is addressed individually in that it first calculates the train’s speed and then uses the result to compute the distance traveled.

Figure 12 is an illustration of least-to-most prompting being applied to a mathematical problem. The initial complex problem is systematically broken down into a series of simpler subproblems. The process begins with the decomposition of the main problem—calculating the distance a train travels in 2.5 h—into two sequential subproblems. First, the model is prompted to determine the speed of the train, and then it uses this information to calculate the distance traveled. Each subproblem is solved in sequence, with the solution to the first subproblem being fed into the second subproblem. The solutions are then aggregated into the final answer. This method emphasizes the key principles of problem decomposition and sequential problem-solving, enabling the model to more effectively manage and solve complex tasks.

Beyond this simple example, the least-to-most prompting paradigm has been shown to be an effective approach for solving complex problems in various domains, including symbolic manipulation, compositional generalization, and mathematical reasoning.^82^

Another example of implementing least-to-most prompting is the Program-Aided Language (PAL)^83^ model, a framework in which LLMs interpret natural language problems and generate executable programs as intermediate reasoning steps. The use of least-to-most prompting has been shown^83^ to improve the performance of PAL on complex mathematical problem benchmarks such as Grade School Math 8K (GSM8K)^84^ and the Simple Variations on Arithmetic Math Word Problem (SVAMP).^85^

### Tree of thoughts

The tree of thoughts (ToT)^86^ prompting technique allows LLMs to explore multiple reasoning paths, called “thoughts,” before producing a final solution. Unlike traditional linear prompts, ToTs allow LLMs to consider various possible solutions and strategies, including looking ahead, backtracking, and self-evaluation, making it more flexible and adaptable to the complexity of the task at hand.^86^

For example, when applied to complex mathematical problem-solving scenarios, the ToT approach prompts the utilized model to generate various potential solutions and evaluate them rather than simply asking for a solution. ToT prompting has been shown to enhance the performance of LLMs by structuring their thought processes.^86^^,^^87^

**Figure 13:**
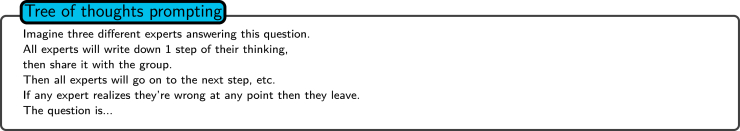
A sample ToT prompt In ToT prompt structure, multiple hypothetical experts iteratively contribute and evaluate each step of their reasoning collaboratively. ToT prompting enables the model to explore different reasoning paths and systematically refine answers by excluding incorrect or less promising solutions at each step.^7^

The ToT prompting^7^ approach assimilates the ToT principles into a streamlined prompting methodology. This technique enables LLMs to assess intermediate cognitive constructs within a singular prompt. Figure 13 provides an example of a ToT prompt.

### Graph of thoughts

The graph of thoughts (GoT)^88^ framework can be viewed as a generalization of the CoT and ToT frameworks. The idea of GoT is to model the information generated by LLMs as an arbitrary graph, which is a more general data structure than a chain or a tree. In this graph, individual units of information, called “LLM thoughts,” are represented as vertices. The edges of the graph represent the dependencies between these vertices.

When addressing a complex challenge, the GoT framework initially produces several autonomous thoughts or solutions from the employed LLM. These individual insights are then interlinked on the basis of their pertinence and interdependencies, leading to a detailed graph. This resulting graph can then be analyzed using diverse traversal methods, which can yield a precise, multifaceted solution to the original challenge.

### Decomposed prompting

Decomposed prompting (DECOMP)^89^ is a modular approach that was designed to address complex tasks by breaking them down into simpler, manageable subtasks, which are then handled by specialized handlers. It can be viewed as a modular extension of the more linear least-to-most prompting technique.

**Figure 14:**
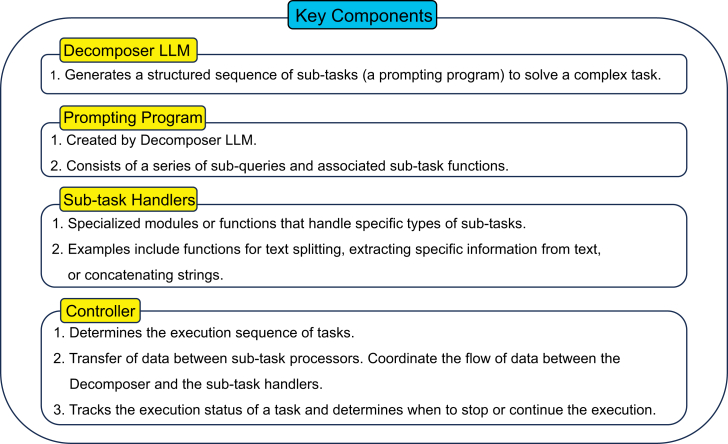
Key components of DECOMP Shown is an overview of the DECOMP framework with four parts: the decomposer LLM generates a structured sequence (prompting program) of subtasks. The prompting programs consist of a series of subqueries and are associated with subtask functions. Subtask handlers are specialized modules or functions. The controller coordinates task execution, manages data transfer, and tracks progress.

**Figure 15:**
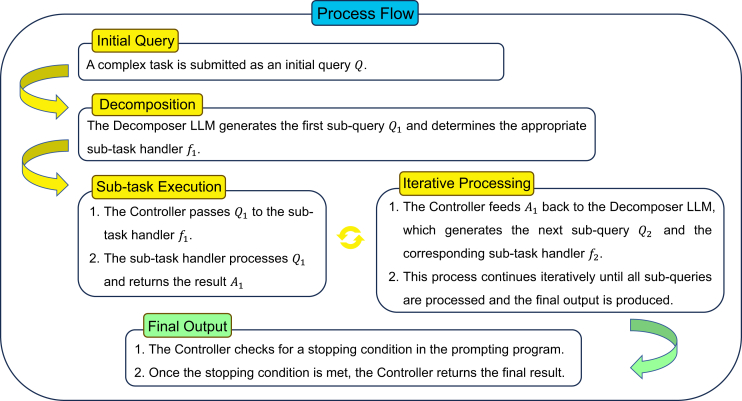
An example of the process flow of DECOMP The process starts when a query is entered. The decomposer LLM breaks down the initial query into sequential subqueries, each handled individually by specialized subtask handlers. The controller manages the iterative process by coordinating between decomposer and handlers. When all conditions are satisfied, the model will provide the final output.

The four key components of this method are shown in Figure 14. The core process of DECOMP involves a decomposer LLM that generates a prompting program *P* for a complex task *Q*. The program *P* is a sequence of steps, with each step directing a simpler subquery to a function within an auxiliary set of subtask functions *F*. The program can be represented as follows:

$$P=\left\{\left(f_{1},Q_{1},A_{1}\right),\ldots ,\left(f_{k},Q_{k},A_{k}\right)\right\}\text{,}$$

where $A_{k}$ is the final answer predicted by *P*, and $Q_{i}$ is a subquery that is directed to the subtask function $f_{i}\in F$. A high-level imperative controller manages the execution of *P*, passing inputs and outputs between the decomposer and subtask handlers until the final output is obtained. In-context examples are used to teach the decomposer LLM. These examples demonstrate the decomposition of complex queries into simpler subqueries. Each example $E_{j}$ takes the following form:

$$E_{j}=\left(Q_{j},\left\{\left(f_{j,1},Q_{j,1},A_{j,1}\right),\ldots ,\left(f_{j,k_{j}},Q_{j,k_{j}},A_{j,k_{j}}\right)\right\}\right)\text{,}$$

where $A_{j,k_{j}}=A_{j}$ is the final answer for $Q_{j}$, and $\left(Q_{j,1},\ldots ,Q_{j,k_{j}}\right)$ represents the decomposition of $Q_{j}$. Each subtask function *f* is operationalized through subtask handlers, which can be additional LLM prompts or symbolic or learned functions.^89^ An illustration of the process flow is shown in Figure 15.

The DECOMP approach has several advantages. First, its modularity allows each subtask handler to be independently optimized, debugged, and upgraded, which facilitates systematic performance improvements and easier integration of new methods or models. Second, DECOMP can incorporate error-correcting subtask handlers, improving the overall accuracy and reliability of the system. Third, the approach allows for diverse decomposition structures, including hierarchical and recursive decompositions, which are particularly useful for handling complex and large-scale problems. Finally, subtask handlers can be shared across different tasks, enhancing the efficiency of the problem-solving process.

DECOMP and least-to-most prompting^82^ both decompose complex tasks to increase the ability of LLMs to execute reasoning tasks, but DECOMP distinguishes itself through its flexible and modular approach. Unlike the linear progression of least-to-most prompting from easy to hard subquestions, DECOMP allows for nonlinear and recursive decomposition with dedicated subtask handlers that can be independently optimized and replaced. This modularity not only enhances the flexibility and reusability achieved across tasks but also introduces potential error-correcting mechanisms, making DECOMP more robust and adaptable to complex, multistep reasoning tasks. Although DECOMP has shown superior performance in specific domains, such as symbolic reasoning and multistep question answering, its advantages over least-to-most prompting may vary depending on the nature of the given task.^89^

DECOMP has demonstrated superior performance in various case studies. For example, in the $k^{th}$ letter concatenation task, DECOMP outperforms CoT prompting by effectively teaching the subtask of extracting the $k^{th}$ letter through further decomposition. In list reversal, DECOMP exhibits better length generalization than CoT prompting by recursively decomposing the task into the reversal of smaller sublists, achieving higher accuracy for longer input sequences. In long-context question answering (QA), DECOMP allows us to handle more examples than CoT prompting can. In open-domain QA, incorporating symbolic retrieval APIs within the DECOMP framework enhances the performance achieved on multihop QA datasets over that attained with CoT prompting. Finally, in math QA, DECOMP improves accuracy by post-processing CoT prompts to fix frequently encountered formatting errors, resulting in significant performance gains.^89^

### Active prompting

**Figure 16:**
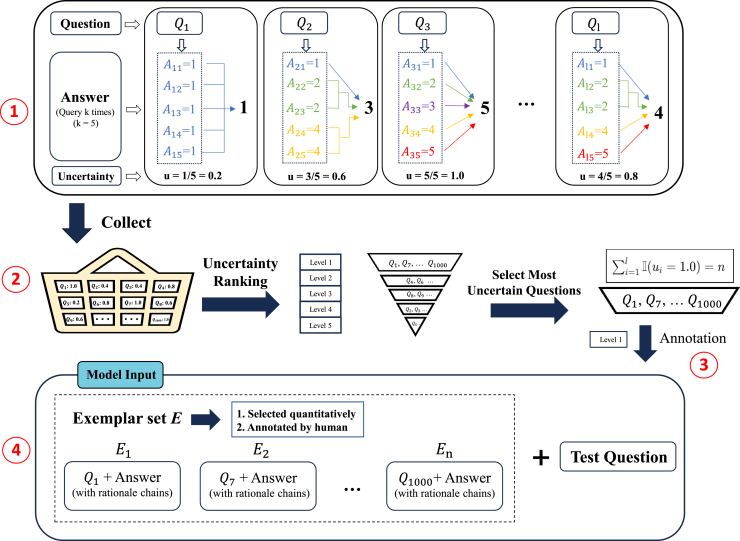
Illustration of the entire active prompting process There are four main stages: (1) the uncertainty estimation by querying the model multiple times per question; (2) collecting, ranking, and selection of the most uncertain questions; (3) annotation of the selected questions by human experts with detailed rationale chains; and (4) final inference with annotated exemplars.

The active prompting^90^ method does not involve the traditional prefix tuning process^91^; instead, it focuses on improving the reasoning capabilities of LLMs through strategic selection and the annotation of task-specific examples. By systematically selecting and annotating the most uncertain questions, this method not only refines the understanding of the employed model but also utilizes human expertise more effectively.^92^ The process begins with the generation of multiple predictions for each question, followed by the calculation of uncertainty (uncertainty estimation)^93^^,^^94^ using various metrics, such as disagreement, entropy, and variance. This strategic selection process ensures that the most informative questions are prioritized for annotation. The human annotation phase is crucial because it involves providing a detailed CoT reasoning procedure and answers, which are then used to prompt the LLM during inference. These annotated data serve as examples, guiding the model through complex reasoning pathways and enhancing its predictive accuracy. The application of self-consistency techniques^26^ can further solidify the reliability of the model by selecting the most consistent answers from multiple reasoning paths. The key innovation of this method is the identification of the most efficient one-shot or few-shot^53^ examples for improving the inference ability of a model in specific fields. An illustration of the concrete process used by this method is shown in Figure 16.

The active prompting method makes the best use of human engineering expertise by focusing on the most uncertain and informative questions, resulting in performance improvements across various reasoning domains. This approach aligns with the broader trend toward more interactive and adaptive AI systems, emphasizing the importance of responsive designs in prompt engineering.^90^

### Prompt pattern catalog

A prompt pattern catalog^95^ is an organized collection of prompt templates and patterns that is designed to simplify prompt engineering in a systematic way. This methodology involves creating a standardized set of prompt patterns that can be applied in various tasks, ensuring consistency and reducing the variability and errors exhibited by ad hoc prompt creation.^95^^,^^96^ Predefined prompt patterns streamline the prompt engineering process by allowing practitioners to select and adapt existing patterns rather than creating new prompts from scratch, saving time and resources, and enabling models to be quickly adapted to new tasks and domains.^97^ The creation of a prompt pattern catalog requires the conceptualization and design of prompt patterns, which can be reused for solving common problems that are faced when interacting with LLMs. These prompt patterns are analogous to design patterns in software engineering and should be properly structured and documented to ensure their adaptability across different domains.^95^

White et al.^95^ detailed the process of constructing a prompt pattern catalog. They systematically categorized prompt patterns into five primary categories: input semantics, output customization, error identification, prompt improvement, and interactions. This classification helped to organize patterns on the basis of their functional roles and the specific problems they address. Then, they introduced a comprehensive catalog of 16 distinct prompt patterns. Each pattern was meticulously documented with the following components: name and classification, intent and context, motivation, structure and key ideas, example implementation, and practical consequences. The prompt patterns covered a wide range of functionalities. For example, the input semantics category includes patterns such as meta language creation, which helps define custom input languages for LLMs. The output customization category features patterns such as output automators and visualization generators, which tailor the generated output to specific formats or visualizations. Error identification patterns, such as fact checking lists, ensure the accuracy of generated content by highlighting critical facts for verification. Prompt improvement patterns, including question refinement and alternative approaches, enhance the quality of interactions by refining questions and suggesting multiple ways to achieve a goal. Finally, interaction patterns, such as flipping interactions and game play, facilitate dynamic and engaging user-LLM interactions.^95^

The prompt pattern catalog methodology encourages the combined use of these standardized patterns to address more complex prompt engineering tasks. White et al.^95^ showed that developing and utilizing a prompt pattern catalog can significantly increase the effectiveness of prompt engineering when working with LLMs such as ChatGPT. In particular, they highlighted the advantages of employing predefined structured prompt patterns in software development tasks, demonstrating substantial improvements in code quality, requirements elicitation, and refactoring efficiency. Similarly, Mondal et al.^97^ examined how predefined structured prompt patterns can be used to enhance user interactions and improve model outputs in conversational AI by consolidating multiple prompts into a more cohesive issue resolution strategy.

### Prompt optimization

To avoid the extensive manual effort and expertise required to craft effective prompts, the idea of prompt optimization involves finding an automatic way to adjust prompts on the basis of their LLM responses, increasing their accuracy and relevance. Several methods have been proposed to automate prompt optimization, including gradient-based approaches, such as prompt optimization with textual gradients (ProTeGi),^98^ which uses text-based gradients to iteratively refine prompts, and black-box methods, which optimize prompts on the basis of output performance without requiring model internals. Furthermore, model-adaptive techniques, such as model-adaptive prompt optimization (MAPO),^99^ tailor the optimization process to the specific characteristics of LLMs, potentially offering superior results. Each method has advantages; gradient-based techniques are efficient and directed, black-box approaches are broadly applicable and easy to implement, and model-adaptive methods provide customized optimization steps for specific models. The selected method depends on the given task requirements, the complexity of the model, and the available resources.

#### ProTeGi

ProTeGi^98^ is inspired by gradient descent, which is a fundamental optimization technique, but it adapts this concept to the discrete text-based nature of NLP. Instead of relying on numerical gradients, ProTeGi generates textual gradients, natural language descriptions of the flaws in a given prompt based on its performance on a small batch of data. These gradients provide semantic guidance on how the prompt should be adjusted to attain improved task performance. The optimization process is further refined through an iterative approach that applies a beam search in combination with a bandit selection strategy to efficiently explore the space of potential prompts and select the most promising candidates for further refinement.^98^

A sentiment analysis example can illustrate the iterative optimization process of ProTeGi. In a sentiment analysis task, ProTeGi begins with an initial prompt that might be too vague for accurately capturing nuanced language. Suppose that the prompt is “Determine whether the following text is positive, negative, or neutral.” When the prompt is evaluated in a small batch of social media posts, ProTeGi identifies specific cases where this prompt leads to incorrect predictions, especially for posts containing sarcasm or mixed sentiments. For example, the model misinterprets the post, “Oh great, another Monday!”, classifying it as neutral rather than negative. ProTeGi addresses this issue by generating a textual gradient natural language feedback indicating that the prompt does not guide the model to recognize sarcasm or subtle cues that can influence the sentiment. On the basis of this feedback, ProTeGi refines the prompt to read “Determine whether the following text is positive, negative, or neutral, paying special attention to sarcasm and subtle language cues that may indicate hidden sentiment.” This modified prompt, which is tested iteratively, leads to improved accuracy in terms of classifying sentiment by making the model more attuned to nuanced language.

ProTeGi has demonstrated strong performance across various NLP tasks, not only in sentiment analysis but also fake news detection and LLM jailbreak detection,^98^ all of which do not require access to the internal workings of LLMs.

#### Black-box prompt optimization

In recent prompt engineering research, the challenge of aligning LLMs with human intentions without additional retraining has garnered significant attention. Traditional methods, such as RLHF and direct preference optimization (DPO) are computationally intensive and inaccessible for closed-source models such as GPT-4 or Claude-2, whose model parameters cannot be easily altered. Instead, black-box prompt optimization (BPO)^100^ refocuses the alignment process from model-centric adjustments to input-centric optimization by refining user prompts instead of altering model parameters. BPO trains a sequence-to-sequence model of feedback-based prompt pairs to iteratively enhance the degree of alignment between prompts and expectations.

An example of BPO involves a user prompt such as “Explain climate change,” which, while straightforward, may elicit a broad or incomplete response. BPO refines this prompt by leveraging human feedback to create a more specific version: “Provide a detailed explanation of climate change, including its causes, major impacts, and solutions for mitigation and adaptation.” This optimized prompt guides the utilized model to produce a more comprehensive response that aligns closely with human expectations of depth and relevance.

The advantages of BPO are threefold. First, BPO is model agnostic and applicable across different LLMs without accessing model internals. Second, it is interpretable, providing transparent and observable prompt modifications. Third, it has demonstrated empirical efficacy, outperforming RLHF and DPO across various models. Moreover, BPO can complement these methods, further improving their outcomes.^100^

#### MAPO

The main principle behind MAPO^99^ is that prompts should be adapted not only to specific tasks but also to the distinct characteristics of different LLMs. Instead of aiming for a one-size-fits-all approach, MAPO is designed to fine-tune prompts to the specificities of individual LLMs, thereby maximizing their effectiveness across various downstream tasks.

MAPO addresses the inherent variability in how different LLMs respond to the same prompt by introducing a two-phase optimization process. The first phase involves establishing a warm-up dataset, where candidate prompts are generated and evaluated for their suitability for each LLM. This is followed by a combination of supervised fine-tuning and reinforcement learning (RL), which involves techniques such as proximal policy optimization and ranking responses from model feedback.

Empirical studies have demonstrated that, compared with conventional task-specific prompt optimization methods, MAPO can provide significantly improved performance in tasks such as QA, classification, and text generation.^99^

#### PromptAgent

The PromptAgent method frames prompt optimization as a strategic planning problem, with its central mechanism being a Monte Carlo tree search (MCTS),^101^ which is a principled planning algorithm that systematically navigates the space of expert-level prompts. Unlike conventional approaches that generate prompts through local variations or heuristic sampling, PromptAgent employs a self-reflective trial-and-error mechanism inspired by human problem-solving strategies. This process enables the utilized model to iteratively refine the given prompts by generating error feedback and using this feedback to simulate and prioritize high-reward paths.

The autonomous incorporation of domain-specific knowledge and detailed task instructions by PromptAgent enables it to perform well across a range of tasks, from general NLP challenges to specialized domains such as biomedical text processing, where precise terminology is crucial. For example, in a biomedical named entity recognition task,^102^ PromptAgent begins with a general prompt to identify diseases in a text. Through iterative feedback and strategic adjustments, the prompt is refined by adding specific guidance to avoid extracting irrelevant biological terms, such as genes or proteins, which are not diseases. This refinement process improves the accuracy of the model, demonstrating the ability of PromptAgent to integrate domain-specific insights through strategic planning and self-reflection.^102^

#### RL

RL for prompt optimization is an advanced technique that was designed to increase the performance of LLMs by iteratively refining the prompts used during the training and inference processes. This method uses the principles of RL to navigate the complex parameter spaces of large models, optimizing prompts for achieving improved task-specific performance. In RL for prompt optimization, a reward function is defined to evaluate the effectiveness of different prompts based on the output of the employed model. The model then uses this feedback to adjust and optimize the prompts through a series of iterations, ensuring that the prompts evolve to maximize the performance achieved for the target task by leveraging the ability of the model to learn from its interactions with the environment.^103^

Consider the task of VQA, where the goal is to generate accurate answers to questions on the basis of visual inputs. When RL is used for prompt optimization, the model can start with a set of initial prompts and iteratively refine them on the basis of the accuracy of the generated answers. For example, if the model is asked “What is the color of the car in the image?” the initial prompts might produce varied responses. A reward function assesses these responses, favoring prompts that lead to correct answers. Over multiple iterations, the model learns to generate more precise prompts, improving its ability to accurately answer similar questions in the future.^104^

#### GPTs (plug-ins)

**Figure 17:**
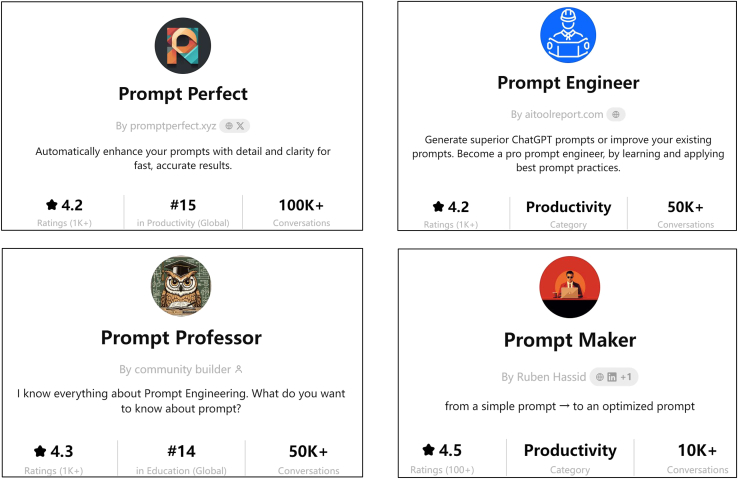
Examples of GPT apps that specialize in generating or optimizing prompts Prompt Perfect is a plug-in that automatically refines prompts for improved performance. Prompt Engineer is an AI assistant to create tailored prompts across creative, technical, and process-driven tasks. Prompt Professor is a tool to improve prompting words on the basis of users’ needs. Prompt Maker is another prompt-assist tool to improve the quality of the prompt.^107^

Before ending this discussion on prompt optimization techniques, we need to mention the use of external prompt engineering assistants that have recently been developed and that exhibit promising potential. Unlike the methods introduced previously, these instruments can help us polish prompts directly. They are adept at analyzing user inputs and subsequently producing pertinent outputs within a context that is defined by itself, thereby amplifying the efficacy of prompts. Some of the plugins provided by the OpenAI GPT store are good examples of such tools.^105^^,^^106^ Some popular GPT store apps that specialize in generating or optimizing prompts are shown in Figure 17.

In certain implementations, the definition of a plug-in is incorporated into the prompt, altering the output.^108^ This integration step may impact the manner in which LLMs interpret and react to prompts, illustrating a connection between prompt engineering and plug-ins. Plug-ins reduce the laborious nature of elaborate prompt engineering, enabling a model to more proficiently address user inquiries without the need for excessively detailed and polished prompts. These tools, which are similar to packages, can be seamlessly integrated into Python and invoked directly.^51^^,^^109^ For example, the Prompt Engineer from GPTs^105^ is a highly versatile AI language assistant that specializes in generating and improving prompts for tasks ranging from creative writing and technical guidance to process optimization. Similarly, another plug-in, called Prompt Perfect, can be used by starting a prompt with “perfect” to automatically enhance the prompt, aiming for the perfect prompt for the task at hand.^110^^,^^111^ Nevertheless, while the use of plug-ins to enhance prompts is a process, it is not always clear which prompt engineering technique or combination of techniques is implemented by a given plug-in due to given the closed-source nature of most plug-ins.

### Retrieval augmentation

Another direction concerning prompt engineering research is to reduce hallucinations. When using AIGC tools such as GPT-4, a problem called hallucinations is commonly faced; this issue refers to the presence of unreal or inaccurate information in the output generated by a model.^22^^,^^112^ Although these outputs may be grammatically correct, they can be inconsistent with facts or lack real-world data support. Hallucinations arise because the model may not have found sufficient evidence in its training data to support its responses, or it may overgeneralize certain patterns when attempting to generate fluent and coherent output.^113^

The idea of retrieval augmentation is to incorporate up-to-date external knowledge into the input of a model to reduce hallucinations.^114^^,^^115^^,^^116^^,^^117^ Ram et al.^118^ showed that directly concatenating relevant information from external sources with a prompt by using autoregressive techniques for retrieval and decoding yields enhanced performance. In another study, Shuster et al.^119^ showed that GPT-3 hallucinations could be reduced by studying various implementations of the retrieval augmentation concept, such as retrieval augmented generation,^114^ fusion-in-decoder,^120^ Seq2seq,^109^^,^^121^^,^^122^ and others. Similarly, the chain of verification^123^ technique was designed to decrease hallucinations by allowing LLMs to deliberate on their initial responses through a process of self-verification and correction. It is suspected that extending this approach with retrieval augmentation would likely lead to further gains. Unified Web-Augmented LLM^124^ converts knowledge-intensive tasks into a unified text-to-text framework and treats the Web as a general source of knowledge.

### Reasoning and active interaction

This subsection explores two advanced techniques that enhance the capabilities of LLMs by integrating reasoning with interaction through external tools or other actions. Automatic reasoning and tool usage (ART) combines CoT prompting with the use of specialized tools. By guiding LLMs through multistep reasoning and incorporating resources such as calculators and databases, ART improves the logical coherence and accuracy of model output. The ReAct (reasoning and acting) framework synergizes reasoning with actionable steps. This prompts LLMs to devise logical sequences and interact dynamically with external tools, enabling them to efficiently handle complex, multistep tasks.

#### ART

ART is an advanced prompting technique that combines the principles of automatic CoT prompting, which encourages models to generate intermediate reasoning steps, with the strategic use of external tools. This method aims to enhance the reasoning capabilities of LLMs by guiding them through multistep reasoning processes and using specialized tools to produce more accurate and relevant output.^125^ This approach helps LLMs handle tasks that require precise calculations, updated information, or complex data-processing steps. For example, a prompt using ART might direct an LLM to outline the steps of a mathematical problem, followed by the use of a calculator to perform the associated computations. This ensures that the outputs are both logically sound and computationally accurate. ART aligns with efforts to develop AI systems that are capable of solving real-world tasks that require a combination of reasoning and computational skills, making it particularly valuable for technical problem-solving tasks such as financial calculations and data analysis.^126^

#### ReAct framework

The ReAct framework,^127^ which has a name standing for “reasoning and acting,” synergizes the processes of reasoning and action to allow LLMs not only to think through problems but also to interact with external tools and environments to produce more accurate and contextually appropriate outcomes. It operates by prompting LLMs to generate both reasoning traces and task-specific actions. This dual approach ensures that the utilized model first considers the given problem, devises a logical sequence of thoughts, and then executes actions that may involve querying external databases, using calculators, or interacting with other software tools. This method is particularly effective in scenarios that require detailed reasoning followed by specific actions, which ensures that the LLM can efficiently handle complex multistep tasks.^127^

**Figure 18:**
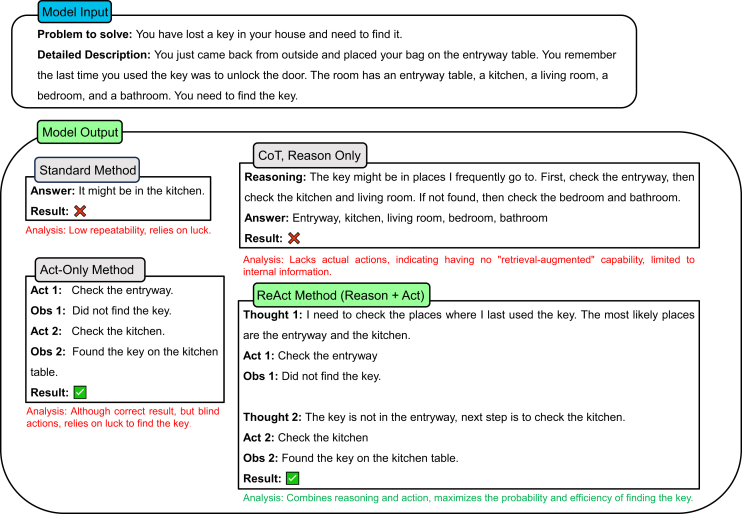
An example of the ReAct method Example of using the ReAct method through a scenario of locating a lost key. It compares standard prompt approaches (reason only and act only) with ReAct’s combined approach. The output shows that the model performs better by integrating step-by-step logical reasoning with sequential actions (“checking entryway, then kitchen”).

For example, in a task that involves financial analysis, the ReAct framework first prompts the LLM to outline the necessary steps for evaluating a portfolio. The model can subsequently use financial analysis tools to gather current market data and perform calculations, integrating these results into the final analysis. This combination of reasoning and action leads to more robust and reliable outcomes than the use of static prompts alone. Another concrete example is shown in Figure 18.

Implementing the ReAct framework is not a trivial task, as it involves developing prompts that guide LLMs through both thought processes and actions. This requires a detailed understanding of the task at hand and the available tools to ensure that the model can seamlessly transition from reasoning to action.

## Methodologies for MMLMs

In recent years, VLMs have made significant developments in multimodal learning by combining visual and linguistic information. These models have demonstrated strong capabilities in tasks such as image description and VQA.^128^^,^^129^^,^^130^^,^^131^ Although this review focuses primarily on the potential for using prompt engineering in LLMs, it is also pertinent to briefly introduce the importance of VLMs and their applications in multimodal tasks to provide a more comprehensive perspective.

VLMs are based on the transformer architecture and are trained on extensive datasets to learn complex semantic relationships. However, unlike early unimodal models, VLMs process both textual and visual information, enabling them to establish connections between image understanding and text generation. As expected, this multimodal integration scheme makes VLMs particularly effective at handling complex tasks that involve both images and text.

To seamlessly integrate and interpret these diverse data types, VLMs require sophisticated prompt designs for ensuring contextual coherence and accuracy.^132^^,^^133^ Challenges such as data alignment, modality integration, and context preservation can be addressed through advanced techniques such as CoOp (see the CoOp section) and MaPLe (see the MaPLe section). These advanced prompt engineering techniques can enhance the ability of VLMs to generate nuanced and contextually rich output, facilitating their effective utilization in various applications.^132^

### Zero-shot and few-shot prompting

Zero-shot and few-shot prompting, which have already been discussed under the section one-shot or few-shot prompting in the context of LLMs, are also pivotal techniques in the realm of VLMs, enabling these models to handle tasks with minimal or no task-specific training data. For example, a model such as CLIP^16^ can be prompted with a textual description to classify images into categories it has never explicitly trained on.^3^ Similarly, few-shot prompting can significantly enhance the ability of a model to generalize with limited data.^16^

In relation to these methods, Awal et al. systematically explored a variety of prompting techniques for conducting zero-shot and few-shot VQA in VLMs, emphasizing the impacts of question templates, image caption integration, and CoT reasoning on model performance.^104^ Radford et al.^16^ demonstrated the effectiveness of these techniques in CLIP, highlighting the ability of the model to generalize across diverse domains by employing natural language supervision. Furthermore, Zhang et al.^134^ presented a method for adapting CLIP to few-shot classification tasks without additional training, emphasizing its practical benefits in real-world applications.

### Continuous prompt vectors

Advances in prompt engineering have enabled more effective adaptations of pretrained VLMs to a wide range of downstream tasks. A promising approach in this domain is the use of continuous prompt vectors to fine-tune models such as CLIP for complex video understanding tasks. Unlike traditional hand-made prompts, which require expert knowledge and manual effort, continuous prompt vectors^135^ are learned during the training process, allowing for a more flexible and efficient model adaptation strategy. This method involves appending or prepending sequences of random vectors to the input text, which the model then interprets as part of its textual input. These vectors are optimized to effectively bridge the gap between static image-based pre-training objectives and the dynamic requirements of video tasks, such as action recognition, action localization, and text-video retrieval. Additionally, lightweight temporal modeling using transformers is applied to capture the temporal dependencies that are inherent in video data.

The efficiency of this approach lies in its minimal computational requirements; only a few parameters are trained, while the core model remains frozen. Despite this, the method has demonstrated competitive performance across various benchmarks, highlighting its potential to extend the capabilities of VLMs for handling resource-intensive video tasks with greater flexibility and accuracy.^135^

### CoOp

CoOp^136^ is a prompt learning approach that was specifically designed for VLMs and is built around the idea of optimizing context-specific prompts. More specifically, CoOp introduces learnable context vectors, which are embedded within the architecture of the utilized model and fine-tuned to minimize the classification loss. CoOp draws on the dual-stream architectures of VLMs, such as CLIP^16^ and A Large-scale ImaGe and Noisy-text embedding,^137^ by performing CoOp on top of these pre-trained models. The learnable context vectors of CoOP can dynamically adjust to different downstream tasks, resulting in better performance and better generalizability in various scenarios.^138^ This method is particularly valuable in applications such as image recognition and VQA, where contexts can vary significantly.^139^

To illustrate a practical application of CoOp, consider a VQA task.^128^^,^^129^^,^^130^^,^^131^ In a VQA scenario, the utilized model is presented with an image and a corresponding question, and it must generate an accurate answer on the basis of visual and textual information. By using CoOp, the model uses learnable context vectors to optimize specific prompts in the context of the input image and question. This process enhances the ability of the model to interpret the visual elements and comprehend the textual query, leading to more precise and contextually relevant answers. For example, if the model is shown an image of a beach scene with the question “What activity are the people engaged in?”, then CoOp would utilize learnable context vectors to help the text encoder generate features that focus on relevant aspects of the image, such as identifying people, recognizing activities, and understanding the overall context of the scene. By aligning these optimized text features with the image features extracted by the image encoder, CoOp can then generate a precise and contextually relevant answer, such as “People are playing volleyball on the beach.”

With respect to the effectiveness of CoOP, Zhou et al.^136^ showed that CoOp-based models significantly outperform traditional models in tasks such as image recognition and VQA. Additionally, Agnolucci et al.^139^ highlighted the benefits of conducting CoOp, which further enhances the performance of the model by combining multiple context vectors. This approach has been shown to improve the robustness and generalizability of VLMs in real-world applications.^140^

### CoCoOp

CoCoOp^141^ is a methodology that dynamically tailors prompts based on specific conditions or contexts. More precisely, CoCoOp employs a lightweight neural network to generate input-conditional prompt vectors for each image while leaving the pre-trained model parameters unchanged. These context-specific prompts generated by the lightweight neural network make it possible to adapt to new and unseen data without the need for fine-tuning the pre-trained model. As a result, a VLM enhanced with conditional prompts can interpret and respond more accurately to images and questions it has not encountered during training.^141^ This capability is critical for applications such as image captioning, VQA, and scene understanding, where contexts can vary widely.

Consider an image captioning task where the goal is to generate descriptive captions for images. By using CoCoOp, a lightweight neural network generates input-conditional tokens that are tailored for different types of scenes, resulting in more accurate and contextually relevant captions. For example, a prompt for an outdoor scene might include contextual cues related to nature, weather, and activities, whereas a prompt for an indoor scene might focus on objects, people, and interactions. For an image of a bustling market, the conditional prompt could include cues such as “Identify the types of products being sold” or “Describe the interactions between vendors and customers.” These cues help the model to produce a relevant caption such as “Vendors selling fresh fruits and vegetables in a crowded market, with customers browsing and purchasing items.”^141^

This dynamic adaptation scheme yields improved caption accuracy and enhances the ability of the model to generalize to novel scenes, addressing the limitations of static prompt methods such as CoOp. In addition to image captioning, the improved generalization capabilities of this technique make the model more robust in tasks such as VQA, image classification, and other real-world applications.^142^

### MaPLe

When facing a multimodal task, prompt engineering techniques generally focus on either visual or linguistic prompts in isolation. In contrast, the core idea of MaPLe is to simultaneously introduce and optimize prompts for both the vision and language components. By embedding prompts at various stages within the transformer architecture, MaPLe ensures that the constructed model can adaptively learn contextual information that is pertinent to the specific task at hand.^143^ The joint optimization of the prompts for both modalities by MaPLe can provide a more coherent representation of the input data, leading to improved performance across a range of applications.^143^

One important aspect of MaPLe is its hierarchical learning mechanism, which allows the model to process and integrate information at multiple levels of abstraction. This is particularly beneficial for complex tasks that require a deep understanding of both visual and textual elements.^143^^,^^144^ As a result, MaPLe has been shown to outperform baseline models such as CoCoOp in tasks including image recognition and VQA.^143^ Table 1 provides a concise comparison between MaPLe and the previously discussed CoOp and CoCoOp methods.

**Table 1:** Comparison of the CoOp, CoCoOp, and MaPLe methods

Method	CoOp	CoCoOp	MaPLe
arXiv submission date	September 2021	March 2022	October 2022
Prompt coupling	none	none	yes (coupling between vision and language prompts)
Fine-tuning CLIP parameters during training	no	no	no
Cross-dataset generalization ability (average over 11 datasets)^143^	63.22	71.69	75.14
Multi-modal data ability	no (optimizes only text prompts)	limited (partially considers both image and text prompts)	yes (explicitly integrates vision and language prompts, enhancing multi-modal understanding)
Advantages	simplifies prompt engineering; performs well on seen classes	dynamic prompts enhance generalization to unseen classes; performs well across tasks and datasets	multi-modal prompt learning and coupling enhance model collaboration and generalization
Disadvantages	static prompts perform poorly on unseen classes, limited generalization; less adaptive to different tasks and datasets	increased computational complexity, potentially requiring more computational resources	more complex implementation, may require more computational resources and training time

To illustrate a practical application of MaPLe, consider the task of VQA.^128^^,^^129^^,^^130^^,^^131^ In a typical VQA scenario, a model is provided with an image and a related question, and it must generate a correct and contextually relevant answer. By using MaPLe, the model can be fine-tuned with multimodal prompts that simultaneously address both the visual content and the textual question. For example, given an image of a bustling market and the question “What fruit is the vendor selling?”, MaPLe embeds prompts at various levels of the vision and language branches of transformer. These prompts might include visual prompts that focus on identifying objects and text prompts that guide the model to look for specific answer-relevant details. By hierarchically processing these prompts, the model can effectively integrate visual cues (such as recognizing apples and oranges in the image) with the textual context (understanding the question) to generate an accurate answer (e.g., “The vendor is selling apples and oranges.”). This multimodal approach ensures that the model taps into both visual and textual information in a coherent and integrated manner, resulting in improved performance for VQA tasks compared with models that do not utilize such comprehensive prompt-learning strategies.

## Assessing the efficacy of prompt methods

There are several ways to evaluate the quality of the output produced by an LLM. The existing evaluation methods can generally be divided into subjective and objective approaches to assess the efficacy of the current prompt methods in AIGC tools.

### Subjective and objective evaluations

The task of prompt engineering can be challenging because it is difficult to determine how effective a prompt is solely on the basis of its raw text form.^145^ Therefore, prompt evaluations require combinations of subjective and objective methods. Subjective evaluations are primarily on the use of human assessors to assess the quality of the generated content. Objective evaluations, which are also known as automatic evaluation methods, either use algorithms to score the quality of the text generated by LLMs or rely on various benchmarks to quantitatively measure the efficacy of prompting methods.

Subjective and objective evaluation methods have advantages and disadvantages. Subjective evaluations are more in line with human intuition, but they are also more expensive and time consuming.^146^ Objective evaluations are less expensive and quicker than subjective evaluations, but they might be less accurate and relevant. Eventually, the best way to evaluate the quality of an LLM output will depend on the constraints and requirements of the specific application of interest.^147^^,^^148^^,^^149^

#### Subjective evaluations

Subjective evaluations depend on human evaluators to judge the quality of generated content. Human evaluators can read the text generated by LLMs and score it according to its quality. Subjective evaluations typically include aspects such as fluency, accuracy, novelty, and relevance.^33^ The chain of density (CoD) technique is a kind of human evaluation method, using a “good summary” standard to assess the effectiveness of the increasingly dense summaries generated by GPT-4.^32^^,^^150^ The four authors of the paper scored 100 summaries that included randomly shuffled CoD summaries to evaluate the performance of their method. Yao et al.^86^ utilized human judgments to compare the outputs of various methods, including the ToT approach, by assessing the ability of the model to complete creative writing tasks. They averaged the scores achieved for each output and reported that the human judgment scores were consistent and therefore credible and reliable. Wang et al.^151^ employed three human annotators to create a dataset aimed at exploring the alignment between human and automated LLM evaluations. Paul et al.^146^ evaluated the quality of generated norms and moral actions using three human judges, who assessed their relevance to the moral story provided.

In addition to these examples, real-world applications of subjective evaluations are exemplified by platforms such as the HuggingFace Chatbot Arena Leaderboard.^152^ This leaderboard uses pairwise comparisons provided by human evaluators to rank conversational AI models, focusing on aspects such as their conversational coherence, contextual relevance, and preference for each output.^153^ While they are primarily focused on conversational AI, such evaluation frameworks provide valuable empirical insights and illustrate how subjective evaluations can quantify the qualitative achieved aspects of model outputs. They complement the theoretical advances in prompt engineering by linking subjective evaluations to measurable, user-driven metrics, offering a practical lens through which the effects of prompt optimization can be evaluated.^153^^,^^154^ Subjective evaluations are being increasingly used to assess the content generated by models in areas that are difficult to represent with datasets and are more abstract, such as writing and summarization.

#### Objective evaluations

Objective evaluations, which are also known as automatic evaluation methods, use algorithms to assess the quality of the content generated by LLMs or to conduct tests on various benchmarks, thereby quantitatively measuring the effectiveness of different prompt methods. The human-AI language-based interaction evaluation method,^155^ which is employed in human-LM interactive systems and evaluation metrics, puts interaction at the center of the LM evaluation. One example of an automated objective evaluation metric is the bilingual evaluation understudy^156^ scoring technique, which assigns a score to system-generated output, offering a convenient and rapid way to compare various systems and monitor their progress. Other evaluations, such as the recall-oriented understudy for gisting evaluation (ROUGE)^157^ and the metric for evaluation of translation with explicit ordering,^158^ assess the similarity between the generated text and the reference text. More recent evaluation methods, such as BERTScore,^159^ aim to conduct assessments at a higher semantic level. However, these automated metrics often do not fully capture the results of the evaluations of human evaluators and therefore should be used with caution.^160^

Many researchers have evaluated their methods by measuring the performance of the constructed models under specific tasks, such as game of 24 and 5 × 5 crosswords,^86^ or benchmarks datasets that contain instructions for the models to complete. Apart from comprehensive sets of benchmarks, such as the “beyond the imitation game benchmark”^161^ and the Big-Bench Hard (BBH) benchmark,^161^ which evaluate the logical soundness of arguments, four main types of benchmarks can be identified, as discussed below. These benchmarks provide standardized tasks and datasets that facilitate consistent and comparable assessments of different approaches.^162^

##### Math word problems

Objective evaluations of a math word problem (MWP) test the ability of a model to understand number-related questions. This task is challenging because the model needs to understand relevant information derived from natural language text and perform mathematical reasoning to solve it. The complexity of MWPs can be measured along multiple axes; e.g., reasoning linguistic complexity and domain knowledge. Similar to the earlier MATH23K benchmark^163^ and the hybrid MWP dataset,^164^ SVAMP^85^ is a kind of MWP benchmark that is used to solve elementary-level MWPs. This benchmark evaluates the performance of models by asking them to provide equations and answers on the basis of elementary school questions. Dolphin1878^165^ is a benchmark containing over 1,500 number-word problems. ARIS^166^ and AllArith^167^ contain arithmetic word problems, and Math Word Problems^168^ contains algebraic word problems that test the problem-solving skills of models. Unlike these benchmarks, which focus on one category or field, Academia Sinica Diverse MWP Dataset (ASDiv),^169^ Algebra QA (AQuA),^170^ and MathQA^171^ gather problems from several areas, such as arithmetic, algebraic, and domain knowledge problems. The singleEQ^172^ method was construed with single-step and multistep math problems from mixed sources. MultiArith^173^ includes elementary math problems with multiple steps. Mathematics Aptitude Test of Heuristics (MATH)^174^ and GSM8K^84^ require models to solve complex mathematical problems, which demands a deep understanding of mathematical concepts and reasoning. Process-supervised Reward Models 800K^175^ includes 4,500 MATH test problems and contains approximately about 800,000 step-level labels over 75,000 solutions.

##### QA tasks

QA tasks require models to return feedback on the basis of the given question. Massive multitask language understanding^176^ is a QA benchmark that was designed to measure knowledge acquired during pre-training by evaluating models exclusively under zero-shot and few-shot settings. Many QA benchmarks are also related to knowledge-based tasks. Fact extraction and verification^177^ focuses on fact verification, which requires models to act on claims generated by altering sentences extracted from Wikipedia. MIDTERMQA^178^ focuses on the 2022 US midterm elections, keeping in mind that the knowledge cutoffs of black-box LLMs are often 2021 or earlier. These benchmarks can play a critical role in assessing the ability of a model to comprehend, analyze, and synthesize information acquired from diverse sources. NarrativeQA^179^ is built upon content material such as movies and books, with nearly 63,000 input tokens of contained input in each question. QA with Long Input Text, Yes (QuALITY)^180^ is a multiple-choice QA dataset that contains 2,000–8,000 tokens collected from English source articles. CommonsenseQA^181^^,^^182^ focuses on commonsense QA tasks implemented via ConceptNet 5.5,^183^ an open multilingual graph of general knowledge. HotPotQA^184^ is collected by crowd-sourcing with data sources such as Wikipedia articles, and the AI2 reasoning challenge^185^ dataset includes 14 million science sentences, 787 science questions, all nondiagram, and multiple-choice questions. The GovReport^186^ dataset focuses on summarizing complex government reports and tests the ability of a model to distill and synthesize critical information. QA benchmarks challenge the reasoning effects of models and their ability to use commonsense knowledge.

##### Language understanding tasks

An example of an early effort that is made to solve language understanding and inductive tasks was the Text Retrieval Conference,^187^ which focuses on the problem of retrieving answers rather than document lists. The Stanford Sentiment Treebank^188^ is constructed with fully labeled parsing trees, enabling comprehensive analysis of the compositional effects of sentiments in language. Summarization tasks, as tested with datasets such as SummScreenFD,^189^ measure the effectiveness of methods in terms of capturing essential information from large amounts of content. AG’s News^190^ is a subset of the larger AG’s Corpus, which is built by compiling titles and description fields from articles belonging to different categories in AG’s Corpus. By pairing the different task instructions with the corresponding text, SentiEval^191^ decreases the sensitivities associated with the prompt design process during the evaluation of different LLMs. Customer Reviews,^192^ which contains the sentiments of sentences mined from customer reviews, and Movie Reviews,^193^ which includes the sentiments of movie review snippets on a five-star scale, are benchmarks that instruct models to classify sentiment from content. “Less likely brainstorming”^194^ is a benchmark that asks a model to generate outputs that humans think are relevant but are less likely to happen. Subj^193^ is a benchmark that includes the subjectivity of sentences derived from movie reviews and plot summaries. Salient long-tail translation error detection (SALTED)^195^ focuses on identifying errors in translations, emphasizing linguistic proficiency and attention to detail. These evaluations highlight the ability of models to understand and process text, yielding accurate predictions based on the given content. The Coin Flip^25^ dataset assesses symbolic reasoning capabilities by asking the evaluated model to answer whether a coin still has its heads facing upward after people either flip or do not flip the coin.

##### Multimodal tasks

Multimodal tasks are designed to evaluate an MMLM’s ability to process and integrate information from multiple modalities, such as text and images. Benchmarks such as Referring Expressions for Common Objects in Context (RefCOCO/RefCOCO+^196^/RefCOCOg^197^) challenge models to identify objects in images on the basis of reference expressions.^198^ For example, RefCOCO tasks might require a model to locate “the red apple on the left” or “the man wearing a blue shirt” within an image. These tasks test not only the ability of the model to understand textual descriptions but also its capacity to map linguistic elements to visual features, demonstrating cross-modal reasoning and comprehension capabilities. Such evaluations are critical for advancing applications including VQA and image captioning, where models must effectively bridge the linguistic and visual domains. Moreover, they provide valuable insights into the impacts of prompt engineering techniques, particularly in terms of designing prompts that guide models toward accurate cross-modal interpretations.

### Comparison among different prompt methods

Some models are used to evaluate the performance of other models.^199^^,^^200^ The performance scores achieved by different methods serve as benchmarks for evaluation. LLM-Eval^201^ was developed to measure open-domain conversations with LLMs. This method aims to evaluate the performance of LLMs on various benchmark datasets,^202^ such as Dynabench,^203^ and demonstrate their efficiency. Many studies^127^^,^^146^^,^^204^^,^^205^ have compared their prompt engineering methods with previously developed prompt methods, such as the CoT, zero-shot, natural instructions,^206^ automatic prompt optimization,^207^ and Automatic Prompt Engineer (APE)^208^ approaches by using benchmarks such as SVAMP,^85^ GSM8K,^84^ ASDiv,^169^ AQuA,^170^ MultiArith,^173^ SingleEQ,^172^ and BBH.^161^ Specific benchmarks are used to test the improvements provided by new prompting methods over the original model. Chen et al.^209^ chose QuALITY, SummScreenFD and GovReport under the original type and long content type to compare their approach with other methods, such as Recurrence^210^^,^^211^^,^^212^ and Retrieval.^120^^,^^213^ Guo et al.^214^ compared their methods with APE and MI^191^ by ROUGE-1, ROUGE-2, and ROUGE-L scores.^157^ Lo et al.^215^ calculated scores via the approach of Yao et al.^216^ and compared them with those of ReAct. The approach of Zhang et al.^217^ performed better than the other tested methods under RefCOCO,^196^ RefCOCO+, and RefCOCOg.^197^

In addition to comparing different methods based on their scores, other indicators can provide additional insights. Jiang et al.^218^ considered the economic costs incurred by various prompt methods. Ning et al.^219^ reported that their skeleton of thought method achieved a significant speedup, often close to twice the original evaluation speed, depending on the model. Li et al.^124^ divided their evaluation into seven domains, such as “dialog,” “slot filling,” and “open-domain QA,” which more comprehensively compared the ability of models to solve tasks. Hu et al.^220^ reported improvements in “accuracy,” “precision,” and “recall” when they compared their chain-of-symbol prompting method with CoT prompting in various spatial reasoning tasks. Feng et al.^178^ evaluated different methods in four categories: “human,” “social,” “STEM,” and “other.”

Subjective comparisons are also used to compare prompting methods. Krishna et al.^221^ introduced the human-rater measure as an evaluation metric. Sun et al.^222^ compared the “planning and executable actions for reasoning over long documents” approach with other methods, such as CoT, program of thought,^223^ Self-Asked,^224^ Toolformer^225^ and ReAct, in four domains: “explicit planning,” “iterative prompting,” “does not rely on external tools,” and “long documents.” Wang et al. combined human and automated evaluations to determine whether their proposed method aligned effectively with human reasoning.^151^ Yao et al.^86^ compared CoT with ToT in human-rated creative writing tasks.

Other studies have focused primarily on certain models or tasks and have employed disparate evaluation metrics, restricting the comparability between their methods.^103^^,^^226^ However, a general evaluation framework called InstructEval,^227^ which enables comprehensive evaluations of prompting techniques in multiple models and tasks, has recently been proposed. InstructEval yielded the following conclusions. In few-shot settings, omitting prompts or using generic task-agnostic prompts tends to outperform the other methods, with the prompts having little impact on the resulting performance; in zero-shot settings, expert-written task-specific prompts could significantly boost the achieved performance, with automated prompts not outperforming simple baselines, and the performance of the automated prompt generation methods was inconsistent, varying across different models and task types, resulting in a lack of generalizability.

## Applications improved by prompt engineering

The output enhancements provided by prompt engineering techniques can make LLMs more applicable to real-world applications. This section briefly discusses the noteworthy applications of prompt engineering in fields such as teaching and programming.

### Assessments of teaching and learning

**Figure 19:**
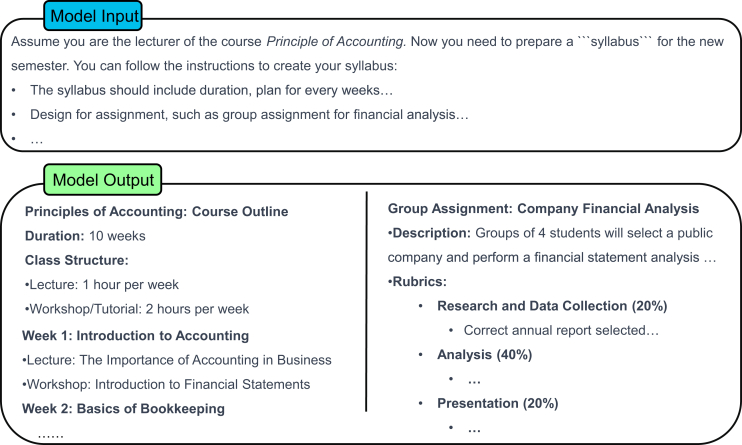
Guidelines of generating a course outline with GPT-4 Example of a course outline generated by GPT-4. With a clear prompt, the model generates an outline that includes weekly class structures with topics and assessment rubrics for group assignments.

In some contexts, prompt engineering can facilitate the creation of personalized learning environments. By offering tailored prompts, LLMs can adapt to the learning pace and style of an individual. Such an approach can allow for personalized assessments and educational content, paving the way for a more individual-centric teaching model. For example, recent advances in prompt engineering have suggested that AI tools can serve students with specific learning needs, fostering inclusivity in education.^228^ As another simple example, it is possible for professors to provide rubrics or guidelines for a future course with the help of AI. As Figure 19 shows, when GPT-4 was required to provide a rubric about a course, through a suitable prompt, it was able to respond with a specific result that may satisfy the imposed requirements.

Advances in prompt engineering also have the potential to enable automated grading in education scenarios. With the help of sophisticated prompts, LLMs can provide preliminary assessments, reducing the workload of educators while providing instant feedback to students.^229^ Similarly, when coupled with well-designed prompts, they can analyze a large amount of assessment data, providing valuable insights into learning patterns and informing educators about areas that require attention or improvement.^230^^,^^231^

### Content creation and editing

Due to their controllable input improvements, LLMs have been primarily used in creative works, such as content creation. The Pathways Language Model (PaLM)^75^ and the prompting approach have been used to facilitate the generation of cross-lingual short stories. The recursive reprompting and revision framework (${\text{Re}}^{3}$)^232^ uses zero-shot prompting^57^ with GPT-3 to craft a story plan that includes elements such as settings, characters, and outlines. It then adopts a recursive technique, dynamically prompting GPT-3 to produce extended story continuation. Another example is detailed outline control (DOC),^233^ which aims to preserve the coherence of a plot across extensive texts generated with the assistance of GPT-3. Unlike ${\text{Re}}^{3}$, DOC employs a detailed outliner and detailed controller for implementation purposes. The detailed outliner initially dissects the overarching outline into subsections through a breadth-first search method, where candidate solutions are generated for these subsections, filtered, and subsequently ranked. This process is similar to the CoT method. Throughout this generation procedure, a detailed open-pre-trained-transformer-based future discriminators for generation^234^ controller plays a crucial role in maintaining relevance.

### Computer programming

Prompt engineering can help LLMs better generate code. For example, the text-to-Structured Query Language model^235^ is able to provide a solution that can be declared correct unless the maximum number of attempts has been reached, using a self-debugging prompting approach^67^ containing simple feedback, unit tests, and code explanation prompt modules. Another example, the multi-turn programming benchmark,^236^ was constructed to implement a program by breaking it into multistep natural language prompts. However, another approach is the repo-level prompt generator,^237^ which can retrieve the relevant repository context and build a prompt for a given task by focusing on code autocompletion tasks. The optimal suitable prompt is selected by a prompt proposal classifier and combined with the default context to generate the final output.

### Reasoning tasks

AIGC tools have demonstrated promising performance in reasoning tasks. Previous research has shown that few-shot prompting can increase the performance of the model in terms of generating accurate reasoning steps for the word-based math problems included in the GSM8K dataset.^26^^,^^65^^,^^75^^,^^84^ The strategy of including reasoning traces in few-shot prompts,^53^ self-talk,^238^ and CoT^25^ has been shown to encourage the developed model to generate verbalized reasoning steps. Uesato et al.^239^ conducted experiments by involving prompting strategies, various fine-tuning techniques, and reranking methods to assess their impacts on enhancing the performance of a base LLM. The authors reported that a customized prompt significantly improved the ability of the model with fine-tuning and demonstrated a significant advantage by generating substantially fewer reasoning errors. In another study, Kojima et al.^57^ observed that solely using zero-shot CoT prompting leads to a significant enhancement in the performance of GPT-3 and PaLM over that of the conventional zero-shot and few-shot prompting methods. This improvement is particularly noticeable when these models are evaluated on the MultiArith^240^ and GSM8K^84^ datasets. Li et al.^241^ also introduced a novel prompting approach called the diverse verifier on reasoning step (DIVERSE). This approach involves the use of a diverse set of prompts for each question and incorporates a trained verifier with an awareness of the reasoning steps. The primary aim of DIVERSE is to increase the performance of GPT-3 on various reasoning benchmarks, including GSM8K. All of these works have shown that, in applications involving reasoning tasks, properly customized prompts can obtain better results from models.

### Dataset generation

LLMs can learn from contextual information, enabling them to effectively generate synthetic datasets for training smaller domain-specific models. Ding et al.^242^ presented three different prompting approaches for generating training data using GPT-3: unlabeled data annotation, training data generation, and assisted training data generation. In addition, Yoo et al.^243^ designed an approach that generates additional synthetic data for classification tasks. GPT-3 is used in conjunction with a prompt that includes real examples from an existing dataset, along with a task specification. The goal is to jointly create synthetic examples and pseudo-labels via this combination of inputs.

### Agents

The emergence of AI-driven agents represents a key development in contemporary AI research, as they have the ability to optimize organizational processes and support sophisticated decision-making tasks across diverse domains.^244^ In this subsection, we distinguish between two categories of agent systems: (1) task-oriented AI agents,^245^ which focus on concrete applications and predefined functionalities, and (2) more generalized agent AI,^246^ which emphasize performing adaptive, multimodal reasoning in complex environments. Prompt engineering underpins both categories by enhancing instruction interpretations, guiding adaptive behaviors, and improving the strategic use of these AI tools. For AI agents, prompt engineering ensures effective task decomposition and tool invocation processes. Agent AI facilitates the orchestration of multimodal inputs, continuous adaptation, and strategic planning, increasing the flexibility and capability of the overall system.

#### AI agents

Recent advances concerning the integration of LLMs with the capabilities of external tools have propelled the development of AI agents.^245^ Systems such as GPT-4 provide plug-in support, which enables functionalities such as internet searching, code execution, and third-party software integration. This exemplifies a transition from simple dialog systems to multifaceted agents that are capable of managing dynamic tasks. Prompt engineering techniques, such as CoT,^25^ can increase the capacity of these agents to decompose complex objectives into manageable subtasks, incorporate user feedback, effectively draw on external services, and disambiguate abstract tasks.^247^

Further innovations, such as planning-guided transformer decoding,^248^ integrate MCTS^101^ with pretrained LLMs to the refine intermediate reasoning steps, improving their autonomy and adaptability, especially in code generation tasks. This synergy underscores how planning algorithms can complement prompt engineering to enhance AI agents. Furthermore, open-source projects such as Auto-GPT^249^ and the Awesome AI Agents repository^250^ provide practical demonstrations of prompt engineering methodologies in multifaceted autonomous environments. For example, Auto-GPT dynamically generates intermediate objectives in response to user input and environmental changes, whereas Awesome AI Agents has diverse applications, including automated market analysis and real-time data summarization tasks. These implementations highlight how prompt engineering provides enhanced adaptability, robustness, and real-world utility in task-specific AI agents.

#### Agent AI

Agent AI^246^ transcends task-specific scenarios, integrating large-scale models, memory systems, planning algorithms, and tool interfaces into cohesive, adaptive frameworks. Prompt engineering guides the interactions among these components, ensuring that they operate synergistically to achieve specified goals.^251^

The evolution of agent AI can be viewed through five perspectives: models, prompt templates, chains, agents, and multi-agents.^252^^,^^253^ Foundational models (e.g., GPT-4) provide the linguistic and reasoning substrates. Prompt templates standardize input structures, ensuring that outputs align closely with the targeted objectives.^95^ The subsequent stages progressively incorporate more complex architectures, culminating in multi-agent ecosystems where autonomous systems collaborate. Throughout this process, prompt engineering remains a central component, shaping the interpretative fidelity of the agent, guiding strategic adjustments, and enabling these systems to operate effectively and self-regulate within dynamic and often uncertain environments. For instance, Chen et al.^254^ introduced AutoAgents, a framework that dynamically generates and coordinates specialized AI agents to solve complex problems. By defining roles, guiding the collaboration process through tailored prompts, and incorporating mechanisms such as adaptive refinement and iterative feedback, AutoAgents enhance the efficiency and coherence of multiagent cooperation.^254^

## LLM security

The rapid iteration of AI models has heightened concerns from the scientific community about their security, particularly as these models increasingly demonstrate capabilities that sometimes surpass human-level performance in a growing number of domains, such as natural language understanding, strategic decision-making, and multimodal reasoning. For example, the “weak-to-strong generalization” research published by the OpenAI alignment team^255^ explored how weak supervision signals can train stronger AI models while also revealing the challenges posed by unintended generalization behaviors under unreliable supervision, which could compromise real-world applications. Similarly, the study “Language Models Can Explain Neurons in Language Models”^256^ emphasizes the importance of transparency for understanding the internal mechanisms of LLMs, providing a foundation for identifying and mitigating potential vulnerabilities. Building on these broader discussions of AI safety and transparency, this section focuses on security challenges that are specific to prompt engineering.

Prompt engineering is crucial not only for optimizing the performance of the models but also for serving as critical components of their security frameworks. By carefully crafting prompts, researchers and developers can identify and help reduce vulnerabilities in LLMs. Effective prompt engineering can expose weaknesses that can be exploited through adversarial attacks, data poisoning, or other malicious activities. Conversely, poorly designed prompts can inadvertently reveal security vulnerabilities in a model^257^; these vulnerabilities could then be exploited by malicious actors, leading to issues such as the disclosure of sensitive information or susceptibility to adversarial attacks. The proactive, open, and in-depth efforts made by researchers to identify and mitigate vulnerabilities through prompt engineering are essential for maintaining the integrity and safety of LLMs in diverse applications.

This is particularly true in critical sectors such as healthcare, finance, and cybersecurity, where prompt attacks against LLMs could lead to significant breaches of sensitive information or disrupt essential services. For example, adversarial attacks can manipulate model outputs to spread harmful or misleading information, whereas data poisoning during training can corrupt the learning process of the utilized model, leading to unreliable output. In finance,^258^^,^^259^ compromised models could result in significant financial losses and undermine trust in automated financial services.^260^

Consequently, continuous research in prompt engineering security to fully realize its benefits and address the emerging challenges is critically needed. A deeper understanding of attack methods and their mechanisms in relation to prompt engineering is essential for enabling both large-model developers and users to better defend against these threats. In this section, we explore several mainstream attack methods that are related to prompt engineering and discuss ways to defend against them.

### Training-phase attacks

Training-phase attacks target the learning process of a model before it is finalized and deployed. By manipulating data, labels, or parameters of the model during training, adversaries can introduce covert malicious behaviors or latent vulnerabilities that may remain undetected until the model deployment stage. Unlike inference-phase attacks, which rely on cleverly crafted input prompts to mislead or extract a deployed model, training-phase attacks embed harmful patterns directly into the foundations of the model. Such exploits can significantly undermine the reliability, security, and generalization capabilities of the model. In the following subsections, we examine two representative training-phase attacks—data poisoning and backdoor attacks—and discuss their impact and associated defense strategies.

#### Data poisoning

Data poisoning attacks involve the injection of malicious or misleading data into the utilized training corpus, corrupting the foundational knowledge of the constructed model before it is deployed. Unlike inference-phase manipulations, which rely on cleverly crafted prompts at runtime, data poisoning surreptitiously alters the internal representations of the model by exploiting vulnerabilities in its data collection or preprocessing pipelines. Once integrated into the large-scale training set of an LLM, poisoned data can guide the model to learn and reproduce inaccuracies, harmful biases, or policy-violating content when prompted.^261^^,^^262^^,^^263^

Recent studies have highlighted the severity of data poisoning in LLM scenarios. Jiang et al.^261^ demonstrated that injecting carefully crafted examples could force generative models to systematically degenerate and produce undesired output, revealing the potential to degrade the quality and trustworthiness of the model.^261^ The PoisonBench framework^262^ further underscores the susceptibility of large models to various poisoning strategies, systematically assessing their vulnerabilities and comparing different attack methods. Although data poisoning inherently occurs during training, its implications directly affect the inference process, making it intertwined with prompt engineering considerations. Effective prompt engineering can help detect anomalies or unexpected model behaviors that may indicate an underlying data poisoning situation. For example, by designing diagnostic prompts and stress-testing LLM responses, professionals can identify suspicious patterns, trace them back to compromised training instances, and implement corrective measures. Furthermore, rigorous data validation steps and robust data governance schemes, such as cross-source verification, data filtering, and provenance tracking, can help prevent the inadvertent inclusion of malicious inputs in the employed training corpus.

The far-reaching consequences of data poisoning extend across multiple sectors, including healthcare, finance, and legal services, where the reliability and accuracy of models are paramount. Although data poisoning presents a complex challenge, research on certified defenses, anomaly detection methods, and secure data pipelines provides pathways for mitigating these risks.^264^^,^^265^^,^^266^ As LLMs continue to scale and are integrated into critical applications, understanding and defending against data poisoning attacks becomes essential for maintaining the integrity, safety, and long-term stability of model deployments.

#### Backdoor attacks

**Figure 20:**
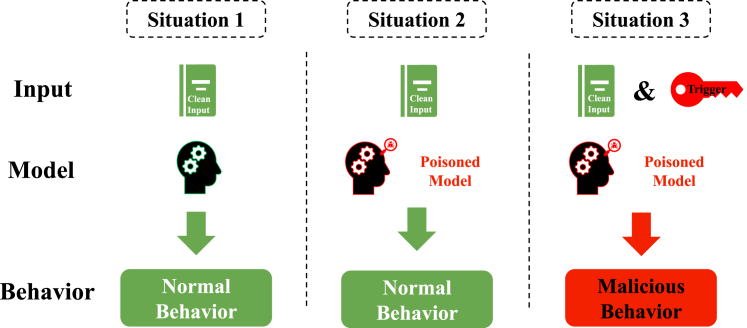
An illustration of three scenarios involving backdoor attacks Shown are three kinds of scenarios in a backdoor attack context: (1) a clean model that produces normal output when given clean input, (2) a poisoned (contaminated) model that produces normal output when receiving clean input, and (3) a poisoned model that produces malicious or incorrect output when triggered by an implanted malicious backdoor signal.

Backdoor attacks involve embedding hidden vulnerabilities within a model during its training phase, which can be activated later during the inference process by specific triggers. Unlike general data poisoning, which broadly alters the decision-making process of the examined model, backdoor attacks rely on carefully planted patterns or “triggers” that lie dormant until a particular prompt or input is encountered.^267^^,^^268^^,^^269^ When presented with this trigger, the model produces predefined, potentially harmful outputs, often without any overt sign of tampering under normal usage conditions. An illustration is shown in Figure 20. Although the malicious effects of backdoor attacks manifest at inference time, when a specific trigger is presented, the core manipulation occurs during training. Hence, we classify backdoor attacks as training-phase attacks with the understanding that their full impact is realized only once the victim model is deployed and queried.

Various studies have explored backdoor attacks in different machine learning (ML) contexts. Early work demonstrated the feasibility of backdooring models through controlled training manipulations,^269^^,^^270^ whereas subsequent research highlighted the complexity of detecting and defending against such threats.^271^^,^^272^ In the context of LLMs, backdoor attacks can be even more insidious, as attackers can exploit prompt engineering to trigger malicious behaviors that bypass alignment and safety measures.^273^

For instance, Gu et al.^267^ introduced the concept of “BadNets” to illustrate how backdoors can be inserted into neural networks along the supply chain.^267^ The reflection backdoor (Refool) approach leverages natural image reflection cues to stealthily implant backdoors into deep neural networks while resisting state-of-the-art defenses.^274^ More recently, Zhao et al.^275^ proposed ProAttack, a clean-label backdoor attack that uses prompts themselves as triggers, eliminating the need for external markers. ProAttack has achieved high success rates in resource-rich and few-shot text classification tasks, revealing critical security vulnerabilities in LLMs and their prompt-based interfaces.^275^

#### Defense and mitigation (training phase)

Training-phase attacks, such as data poisoning and backdoor attacks, present complex challenges because they embed malicious patterns directly into the foundations of a model. Defenses at this stage must operate before the model deployment stage, focusing both on the integrity of the training data and the internal consistency of the model. Although these two attack forms differ in their activation mechanisms—data poisoning broadly skews model behaviors, whereas backdoors lie dormant until they are triggered during the inference process—they share the fundamental threat of covertly compromising the reliability, security, and trustworthiness of the attacked model.

##### Data poisoning

Data poisoning defenses revolve primarily around ensuring the quality and authenticity of the given training corpus. One key approach is data sanitization and filtering, which involves implementing strict data vetting processes, such as cross-source verification and data provenance,^276^^,^^277^ to reduce the probability of malicious samples entering the constructed dataset.^266^ Another line of defense comes from certified defenses and robust training algorithms, which provide formal guarantees and rely on optimization techniques that minimize the sensitivity of the utilized model to a small subset of manipulated points.^264^ In addition, performing anomaly detection within the training set through the use of spectral methods, statistical anomaly detection, or clustering-based inspections can identify outliers that signal potential poisoning attempts. By removing or reweighting suspicious samples, practitioners maintain the integrity of their datasets and the fidelity of their models.^263^^,^^278^

##### Backdoor attacks

Backdoor defenses must detect and neutralize hidden triggers that are embedded during training. Model inspection and trigger detection techniques, such as neural cleansing^279^ and universal litmus patterns (ULPs), analyze trained models for suspicious activation patterns, with ULPs offering a computationally efficient alternative by using optimized input patterns to rapidly detect backdoor-infected models. Runtime methods such as STRong Intentional Perturbation (STRIP)^280^ detect backdoors by injecting intentional input perturbations and observing whether minimal prediction randomness indicates potential trigger inputs. Beyond detection, fine-grained model auditing and model editing techniques^281^^,^^282^ allow practitioners to pinpoint and remove suspicious pathways in the internal representations of the model. Research on fine-pruning^283^ has demonstrated a hybrid approach that combines neuron pruning and fine-tuning to effectively neutralize backdoors, achieving high success against both baseline and pruning-aware attacks with minimal impact on the accuracy achieved for clean inputs. Finally, ensuring a diverse and representative training set and employing robust preprocessing steps can reduce the ability of an attacker to implant a stealthy backdoor trigger that remains undetected until the inference phase.^263^^,^^284^

While data poisoning and backdoor attacks differ in their activation conditions (data poisoning broadly changes the behavior of a model, whereas backdoors rely on specific triggers), some defenses work against both types of attacks. For example, they both benefit from rigorous data governance, including provenance tracking and verification. Typically, stress-testing models with diagnostic prompts and adversarial samples enables the detection of subtle behavioral anomalies that may indicate a poisoning attack or a hidden backdoor. Additionally, community-driven benchmarking and standardized testing scenarios foster a shared understanding of attack vectors and increase the efficacy of defenses,^285^ guiding practitioners toward more secure model development pipelines. In essence, defending against training-phase attacks requires a proactive stance that combines data quality assurance, robust optimization techniques, model auditing, and so on. This multi-pronged approach lays a more secure model foundation, reducing the risk that adversaries will successfully embed malicious patterns that only surface once the model is operational. Table 2 provides a concise overview of these defenses.

**Table 2:** Defense and mitigation for addressing training-phase attacks

Attack type	Defense strategy	Objective	Key techniques
Data poisoning	data sanitization and filtering	ensure the integrity and authenticity of datasets	cross-source verification, data provenance tracking, strict preprocessing
	certified defenses and robust training algorithms	provide formal resilience against manipulations	optimization techniques that minimize sensitivity to corrupted samples
	anomaly detection within the training set	identify and remove suspicious data instances	spectral methods, statistical anomaly detection, clustering-based inspections
Backdoor attacks	model inspection and trigger detection	detect hidden triggers before deployment	neural cleanse, ULPs, STRIP
	fine-grained model auditing and model editing	remove embedded malicious pathways	post-training auditing, parameter editing (e.g., BadEdit)
	diverse, representative training data and robust preprocessing	reduce the attacker’s ability to implant stealthy triggers	rich, vetted datasets; careful data screening
Both	integrated approaches	enhance the overall trustworthiness of model	rigorous data governance, diagnostic prompts, community-driven benchmarks

### Inference-phase attacks

The inference phase provides attackers with direct interaction points through which they can manipulate the behavior of an LLM at runtime. Unlike training-phase attacks, which embed malicious patterns into the parameters of the target model, inference-phase attacks operate by introducing carefully crafted inputs that exploit the decision-making vulnerabilities of the model in real time. These methods often require minimal resources but can circumvent established safeguards, leading to undesirable results, such as harmful misinformation or the unintended disclosures of sensitive details.^286^^,^^287^^,^^288^ In the following subsections, we examine several key categories of inference-phase attacks and discuss their implications for prompt engineering and model deployment.

#### Prompt-level adversarial attacks

Prompt-level adversarial attacks target the model inference process by injecting carefully crafted textual cues into the input, inducing the target LLM to produce undesired or misleading output without altering its underlying parameters.^286^^,^^287^ Such attacks are partial extensions of traditional adversarial example generation techniques to the realm of LLM-based language understanding; they rely on subtle perturbations or universal triggers that steer the reasoning process of the model away from its intended behavior.^50^^,^^289^^,^^290^ This approach can allow attackers to control the model responses in real time, posing significant challenges for both prompt engineering and downstream applications.

**Figure 21:**
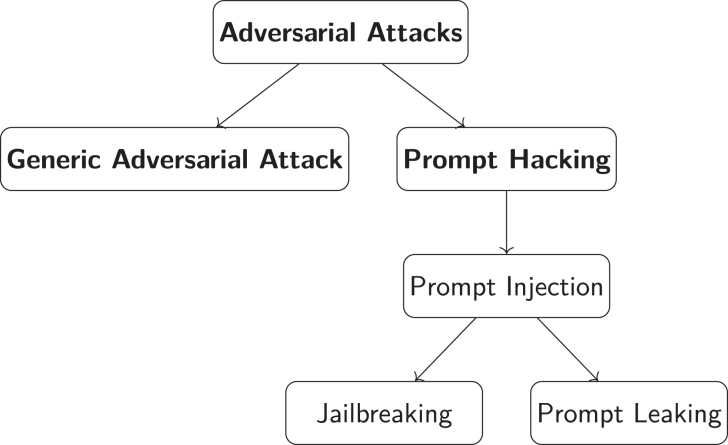
Hierarchical classification of adversarial attacks The primary attack methods can be categorized into generic adversarial attacks and prompt hacking. For prompt hacking, there are several subtypes: prompt injection with jailbreaking and prompt leaking.

Recent research has explored various techniques for conducting adversarial attacks on LLMs. For example, Wang et al.^291^ showed that adversarial demonstration attacks can effectively manipulate erroneous outputs in various scenarios and verified that incorrect predictions are caused by changes in the input data and not the inherent randomness of models. Similarly, Zou et al.^292^ proposed universal adversarial suffixes that exhibit high transferability across different LLMs, including GPT-3.5 and GPT-4, revealing vulnerabilities in model alignment mechanisms. In addition to these two examples, Shayegani et al.^293^ conducted a comprehensive survey of adversarial attacks on LLMs, categorizing various attack types and discussing the roles of optimization and automation techniques in enhancing their effectiveness. Their work revealed critical vulnerabilities in LLMs, particularly in safety-aligned models, including gaps in training by which the models fail to address complex or obfuscated attack scenarios, and underscored the increasing challenges associated with developing robust defenses. Similarly, Liu and Hu^266^ reviewed security vulnerabilities in LLMs with a particular emphasis on prompt hacking and adversarial attacks and explored various defense mechanisms. Figure 21 illustrates the hierarchy structure followed by the primary adversarial attack methods, each of which is subsequently examined in detail in the following subsections.

##### Generic prompt-level adversarial attacks

**Figure 22:**
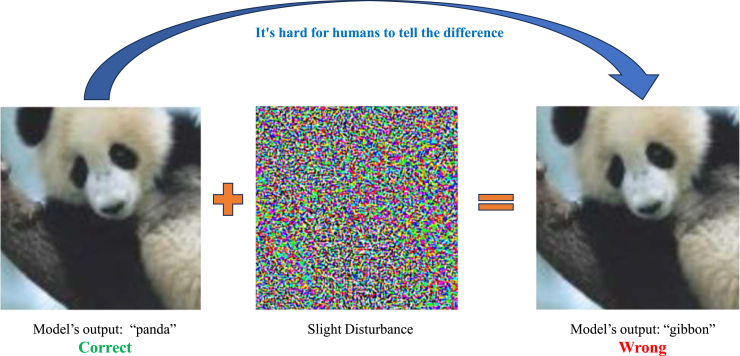
An example of an adversarial attack that misleads a model Example of an adversarial attack on an image recognition model where an input image with slight disturbances misleads the model into incorrect classification: identifying a panda as a gibbon.

In the context of LLMs, these attacks can take the form of subtly altered prompts or inputs that cause the target model to produce unwanted, biased, or harmful outputs. This manipulation exploits the sensitivity of LLMs to small perturbations in the input data, revealing significant vulnerabilities.^286^^,^^294^^,^^295^^,^^296^^,^^297^ For example, in legal document analysis tasks, adversarial inputs can lead to incorrect legal interpretations, potentially affecting case outcomes. In automated healthcare customer service, such attacks could mislead models into providing incorrect medical advice, jeopardizing patient safety.^298^ One example of an adversarial attack in the image recognition field is illustrated in Figure 22.^50^^,^^299^ These examples highlight the need for effective defenses against adversarial attacks to ensure the safe and reliable deployment of LLMs for applications in which the integrity and accuracy of their responses are critical.

##### Prompt hacking and its variants

Prompt hacking refers to a class of attacks that involve manipulating the input prompts provided to LLMs with the goal of provoking unintended behaviors ranging from benign errors to severe consequences, such as dissemination of misinformation or data breaches. Prompt hacking exploits the fundamental way in which LLMs process and generate responses. Unlike traditional hacking, which exploits software vulnerabilities, prompt hacking relies on strategically crafting malicious inputs to deceive an LLM into performing actions that deviate from its intended function.^266^^,^^300^^,^^301^^,^^302^^,^^303^ This vulnerability is particularly concerning because it can be executed without the need for sophisticated technical skills. As LLMs become more integrated into various applications, the risk posed by prompt hacking increases, necessitating robust security measures for preventing such attacks.^304^ Prompt hacking has received increasing attention in academic circles; for example, Schulhoff et al.^305^ presented a large-scale study on the vulnerabilities of LLMs to prompt injection attacks by organizing a global prompt hacking competition, which resulted in the creation of a large dataset and a comprehensive taxonomy of adversarial prompt types.^305^

Prompt injection: Prompt injection attacks craft malicious instructions or contextual cues within the user’s input prompt, causing the employed LLM to generate harmful, misleading, or disallowed information. These attacks exploit the inherent susceptibility of an LLM to adversarial examples,^50^ extending the concept beyond the original domain of computer vision to sophisticated language manipulation. For example, subtle triggers at the prompt level could bypass alignment objectives or built-in safety filters, leading the target model to reveal policy-violating output.^287^ Prompt injection represents a low-cost real-time strategy through which adversaries can manipulate LLM responses without modifying the underlying model parameters. Liu et al.^306^ systematically investigated prompt injection attacks on LLM-integrated applications and proposed the HOUYI method to demonstrate the vulnerabilities of the model and highlight the need for robust defenses against these emerging threats.^306^

(1) Jailbreaking attacks: Jailbreaking attacks represent a specialized form of prompt injection that was designed explicitly to circumvent the alignment policies and content restrictions implemented within LLMs. Unlike generic prompt manipulations that might merely nudge a model toward undesired outputs, jailbreaking aims to break through predefined safety measures, enabling the generation of content that the model is nominally configured to avoid, such as disallowed, harmful, or misinformative material. These attacks often exploit the adherence of a model to role instructions, conditional logic, or internal knowledge representations, prompting the LLM to violate its own guardrails.^287^^,^^307^^,^^308^ As a result, jailbreaking exposes vulnerabilities in the enforcement of rules and content filters by the model, posing significant deployment and policy compliance challenges. Recent analyses have shown that even aligned language models, which have undergone extensive safety training, remain susceptible to jailbreaking attempts.^257^^,^^292^ Furthermore, Wei et al.^309^ identified and analyzed the failure modes in LLM safety training processes, introducing novel jailbreaking attacks that exploit competing objectives and mismatched generalization, demonstrating persistent vulnerabilities in the state-of-the-art safety-aligned models.^309^ All of these studies highlight the need for more robust and context-sensitive defense mechanisms in production environments.
(2) Prompt leaking: Prompt leaking focuses on extracting sensitive or proprietary information embedded within prompts or the context of a model. Unlike traditional adversarial attacks, which aim to produce harmful outputs directly, prompt leaking is concerned with harvesting private data a model might possess or infer. Research has indicated that certain probing prompts can induce LLMs to reveal their training details or confidential content,^310^ raising significant security and privacy concerns. Such vulnerabilities are not purely hypothetical; recent surveys^285^ highlighted prompt leaking as a tangible threat requiring careful data governance, controlled prompt formatting, and robust access control mechanisms to prevent inadvertent disclosure.

To better contextualize prompt leakage and the related threats, we compare them with generic prompt-level adversarial techniques. Table 3 provides a concise overview of these attacks, summarizing their goals, key characteristics, and affected policies or guardrails.

**Table 3:** Comparison of prompt-level adversarial attacks and their variants

Attack type	Goal	Key mechanisms	Affected policies or guardrails
Generic prompt-level adversarial examples	misleading or undesired outputs	subtle textual perturbations that exploit linguistic sensitivities of models	none or minimal policy-targets; mainly exploit model fragility
Prompt hacking (umbrella concept)	broad manipulation of model behaviors via prompts	range of techniques that rewrite, inject, or reshape prompts to produce harmful or restricted outputs	potentially all: alignment constraints, content filters
Prompt injection	bypassing restrictions and inserting hidden instructions	embeds malicious instructions to override content filters or policies of the models	content filters, role instructions
Jailbreaking	overcoming alignment and content filters to produce originally disallowed outputs	targets the policy boundaries of models to unlock restricted content	alignment policies, content moderation layers
Prompt leaking	revealing proprietary data, internal instructions, or other confidential context intended for internal use only.	crafted prompts induce the disclosure of proprietary data or training set secrets	confidentiality policies, access controls

#### Model stealing

Model stealing attacks represent a class of inference-phase strategies that take advantage of prompt engineering techniques to replicate functionality or extract proprietary knowledge from LLMs. Unlike prompt-level adversarial attacks, which primarily aim to induce undesired or harmful outputs, model stealing focuses on reconstructing the underlying capabilities of the model, decision boundaries, or training data distributions of the target model.^311^ This difference in objectives is crucial; while prompt-level adversarial attacks seek to manipulate the immediate responses of the model, model stealing endeavors to reimplement or clone high-value models without direct access to their architectures or parameters.

By systematically designing a diverse set of prompts that cover a broad input space, adversaries can approximate the behavior of a model with a surrogate that closely mimics the target output. Early model extraction studies focused on traditional ML models and APIs,^312^ revealing that carefully chosen queries can uncover learned representations and sensitive information.^313^^,^^314^^,^^315^^,^^316^ As LLMs grow more capable and are widely deployed via cloud-based APIs, attackers can exploit prompt manipulation not only to produce disallowed content but also to investigate the hidden capabilities, alignment constraints, and proprietary training data of these models.

For example, Carlini et al.^311^ successfully extracted parts of production-level language models, underscoring the severity of these threats and the urgent need for robust defenses.^311^ The success of model stealing can lead to intellectual property theft, the erosion of competitive advantages, and the unethical deployment of cloned models in unauthorized contexts.^317^^,^^318^ This increasingly realistic threat scenario calls for a combination of measures, such as improved access control schemes, rate limiting, output obfuscation, watermarking, and more sophisticated prompt-level defenses.

#### Defense and mitigation (inference phase)

The mitigation of inference-phase attacks requires a multifaceted approach that balances usability, model performance, and robust security measures. Unlike training-phase interventions, inference-phase defenses must operate in real time, detecting and neutralizing malicious prompts or inputs before they guide the model to produce unsafe content or leak sensitive information.

The first line of defense involves the use of stricter prompt validation and sanitation techniques. By automatically detecting suspicious patterns, such as hidden instructions, adversarial triggers, or requests for prohibited information, in incoming prompts, systems can preemptively block or review queries that can induce unwanted model behaviors.^257^^,^^301^ In addition, heuristic or rule-based filters can flag requests that deviate significantly from normal usage patterns, whereas anomaly detection models can learn representations of benign commands to identify malicious outliers.^266^

Another effective strategy is to incorporate layered content moderation and alignment reinforcement schemes in the inference stage. This can involve applying dynamic reranking or reverification steps to the raw outputs of a model before finalizing the responses. For example, employing a secondary model or policy-checking mechanism to ensure compliance with the imposed alignment constraints and to block disallowed content can counteract adversarial manipulations employed at the prompt level, including jailbreaking attacks.^287^^,^^309^

The generation of adversarial examples involves creating subtly perturbed inputs that appear normal to humans but can mislead a model. These can then be used to uncover and mitigate vulnerabilities.^50^^,^^289^^,^^290^ Indeed, adversarial training, which involves training models on adversarial examples, has proven effective for enhancing the robustness of LLMs.^319^^,^^320^^,^^321^^,^^322^ This method improves the resilience of models against attacks and enhances their overall reliability. To maximize the effectiveness of adversarial training, the integration of a robust prompt design plays a critical role. The robust prompt design process involves systematically crafting prompts that challenge the model by simulating adversarial conditions, enhancing its ability to generalize and resist adversarial attacks. These techniques not only help evaluate the robustness of the model but also provide targeted feedback to enhance its adversarial learning process.^323^ In some cases, conducting adversarial training or fine-tuning against known prompt attack exemplars may harden the internal representations of the model, making it less susceptible to subtle textual triggers and policy-bypassing attempts.^292^^,^^293^

Access control measures, such as rate limitations and authentication schemes, can also help prevent systematic prompt probing and leakage attacks by ensuring that high-stakes deployments incorporate robust auditing and that logging systems can deter persistent attackers by enabling post-incident analysis and prompt refinement steps.^285^ In addition, community-driven efforts, such as the Open Worldwide Application Security Project (OWASP) LLM prompt hacking project,^324^ provide developers and security professionals with best practices, standardized testing scenarios, and training resources that enable them to strengthen their defense strategies.^324^ Table 4 provides a concise overview of these defenses and their applicability.

**Table 4:** Defense and mitigation for addressing inference-phase attacks

Defense strategy	Objective	Key techniques	Examples/applications
Stricter prompt validation and sanitation	detect and neutralize malicious prompts	automated screening for hidden instructions, adversarial triggers, and prohibited information	blocking harmful queries in real-time applications
Heuristic and rule-based filters	flag and block anomalous requests	predefined rules and heuristics for identifying abnormal prompt structures or content	customer service bots, healthcare advice systems
Anomaly detection models	identify outlier prompts indicating potential attacks	ML models trained on benign prompts to detect deviations	monitoring user inputs for abnormal patterns
Layered content moderation and alignment reinforcement	ensure outputs adhere to ethical guidelines and safety policies	dynamic reranking, secondary verification, policy enforcement mechanisms	multistep content approval processes
Adversarial training	enhance model robustness against adversarial inputs	training with adversarial examples, integrating robust prompt designs, and fine-tuning with prompt attack exemplars	fine-tuning LLMs with diverse adversarial prompts
Access control measures	prevent systematic probing and leaking attacks	rate limiting, authentication, robust auditing, logging	securing high-stakes deployments
Community-driven efforts	provide best practices and standardized testing scenarios	prompt hacking project, developer guidelines, security resource sharing	OWASP collaborative security frameworks

### Evaluation and benchmarking

As the landscape of LLM security threats diversifies, from data poisoning and backdoor attacks to adversarial manipulations at the prompt level and model theft, a critical challenge lies in systematically evaluating and benchmarking the effectiveness of defenses and the resilience of LLMs under adversarial conditions. Reliable evaluation frameworks and standardized benchmarks are essential for quantifying vulnerabilities, comparing defense strategies, and guiding practitioners toward more robust deployments.

An LLM security evaluation needs to consider both the training and inference phases. For training-phase attacks, tools such as PoisonBench^262^ provide a structured environment in which the susceptibility of large models to data poisoning attacks can be assessed during the preference learning stage. This framework systematically evaluates two poisoning strategies, content injection and alignment deterioration, revealing their impacts on the behaviors and alignment objectives of models. While PoisonBench does not directly implement defenses, it serves as a solid benchmark for supporting the development of defenses. Similarly, the theoretical framework of certified defenses against data poisoning attacks^264^ provides valuable information about the worst-case impacts of attacks, which can inform anomaly detection methods and data governance strategies. For backdoor attacks, empirical evaluations often involve curated datasets with known triggers and established detection protocols (e.g., neural cleanse^279^ and ULPs^325^) that test the sensitivity of models and defenses to hidden activation.

In the inference-phase context, the evaluation paradigm shifts toward assessing the real-time robustness of the utilized model. Open-source initiatives such as the OWASP LLM prompt hacking project^324^ encourage community-driven testing scenarios that emulate prompt-level adversarial attacks, prompt injections, and jailbreaking attempts. Complementary efforts, such as OpenAI Evals^326^ and standardized evaluation suites for toxicity, factuality, and policy compliance (e.g., RealToxicityPrompts^327^), allow researchers to investigate models for determining alignment failures and undesired output triggered by adversarial prompts. Moreover, universal adversarial triggers and suffixes^286^^,^^292^ provide test beds for investigating how different LLMs architectures withstand sophisticated inference-time manipulations.

Holistic frameworks, such as Holistic Evaluation of Language Models (HELM),^328^ offer a multidimensional approach for assessing the performance and robustness of LLMs. Although it was originally designed for general capability and bias evaluations, the extensible framework of HELM can incorporate security-focused scenarios, measuring the vulnerability of models under adversarially crafted inputs and the effectiveness of applied defenses. By integrating specialized security benchmarks with broader evaluation environments, researchers could better contextualize security performance relative to other performance axes, such as general reasoning capability, factual correctness, and fairness.

Furthermore, since many evaluation frameworks rely on controlled prompt variations to test model vulnerabilities, they intrinsically measure the effectiveness of prompt engineering strategies to mitigate these threats. By incorporating adversarial prompts or diagnostic scenarios, practitioners can directly link improvements in security metrics to enhancements in prompt engineering designs, ensuring that ongoing efforts to “unleash the potential of prompt engineering” are grounded in systematic, evidence-based assessments.

Another key direction concerns the development of reproducible, transparent, and community-driven benchmarks that evolve alongside the threat landscape. As novel attacks (e.g., prompt stealing^318^^,^^329^ or advanced model extraction techniques^317^^,^^330^) surface, adaptively updating benchmarks ensures that defenses remain tested against state-of-the-art adversarial methods. Initiatives that openly share code, datasets, adversarial triggers, and evaluation metrics accelerate the refinement of security strategies, fostering a collaborative ecosystem of researchers, practitioners, and industry stakeholders.^285^

### Conclusion and prospects for LLM security

While the previous sections delineated various training-phase and inference-phase attacks along with their corresponding defense mechanisms, the reality of secure LLM deployment demands an integrated, adaptive, and forward-looking strategy. Ensuring robust security involves not merely applying isolated countermeasures but also orchestrating them in a comprehensive, in-depth defense architecture. For example, sound data governance and anomaly detection processes in the training stage can reduce the risk of data poisoning or backdoors, whereas prompt validation, dynamic policy checks, and adversarially trained models reinforce defenses against inference-phase exploits.

In the future, fostering a more cohesive security ecosystem for LLMs will hinge on several key directions. First, developing standardized benchmarks and community-driven evaluation frameworks is crucial for systematically comparing defense methods, identifying weaknesses, and promoting best practices.^285^ Second, deeper insight into LLM internals, enabled by advances in model interpretability and neuron-level explanations, can guide the creation of more targeted and context-sensitive defenses that address subtle vulnerabilities. Third, as LLMs continue to be integrated into high-stakes domains such as healthcare, finance, and critical infrastructures, interdisciplinary collaboration will be essential, bringing together expertise from AI, cybersecurity, policy-making, and industry stakeholders.

## Prospects

As LLMs rapidly advance, prompt engineering stands at a critical juncture. While the existing techniques have significantly improved model performance across various tasks, fundamental challenges remain with respect to interpretability, semantic grounding, and the robust application of LLMs in complex real-world scenarios. Addressing these challenges requires looking beyond the current methods and exploring deeper aspects of the architectures, reasoning processes, and semantic understanding capabilities of models. This section outlines two key trajectories for the future of prompt engineering research: gaining a more comprehensive understanding of model structures and their internal dynamics and ensuring that performance improvements are matched by genuine semantic comprehension, leading to more transparent, reliable, and semantically grounded LLM-based systems.

### A better understanding of model structures

A key future direction for prompt engineering research is to achieve a deeper understanding of the underlying structures of LLMs. Models such as GPT-4o^17^ are composed of intricate layers, attention heads, and parameter distributions that shape how they process and generate language. Gaining insights into these architectural components could guide the design of prompts that more effectively engage the internal mechanisms of the models, producing outputs that align more closely with the intent of users.^331^

Recent methodological advances offer promising avenues for probing these internal dynamics. For example, activation patching^332^ introduces a framework that analyzes the behaviors of LMs via specially designed pairs of prompts (e.g., “clean” vs. “corrupted”). By comparing activation induced under different prompt conditions, researchers can pinpoint where and how internal state changes lead to particular output. This can reveal a clearer map of the “decision landscape” of the model and enable the refinement of prompt strategies on the basis of known structural sensitivities.

Furthermore, deeper structural insights could help us understand the limitations of the current prompt methodologies. Webson et al.^332^ have found evidence of limitations in previous prompt models and have questioned how much these methods truly understood the model.^21^ On the other hand, innovations such as the “causal transformer”^333^ explicitly encode causal relationships, offering a blueprint for architectures that may be more transparent and, therefore, easier to steer via prompts.

A more profound grasp of model structures aligns closely with the goals of explainable AI,^334^ ultimately fostering greater trust in LLM applications. In finance, where models are increasingly employed for fraud detection, risk assessment, and compliance, transparency and interpretability are paramount.^335^^,^^336^ Similarly, in healthcare, explainability is crucial for ensuring that AI-driven diagnostic tools and treatment recommendations are reliable and ethically deployable.^337^^,^^338^ Drawing on architectural insights, reproducibility research, and interpretability tools, such as activation patching, future prompt engineering efforts can move beyond surface-level improvements. Exploiting a comprehensive understanding of model structures could set the stage for addressing more subtle, foundational challenges, such as semantic grounding, which will be discussed in the subsequent section, and pave the way for LLMs that are not only accurate and efficient but also inherently trustworthy, interpretable, and robust in real-world scenarios.

### Toward semantic understanding

Recent advances have propelled models such as GPT-o1 Pro Mode^339^^,^^340^^,^^341^ and Claude-3.5 Sonnet^8^^,^^9^ to achieve remarkable increases in their reasoning complexity, context handling capabilities, and overall task performance. These state-of-the-art systems can maintain extended reasoning chains, produce contextually coherent responses, and solve problems in ways that appear increasingly human like. However, enhanced performance and more complicated reasoning pathways do not inherently confirm that these models understand the semantics of the language and concepts they manipulate. Instead, as previous research has suggested,^21^^,^^342^ much of their ability may still be dependent on sophisticated pattern recognition techniques rather than genuine semantic comprehension.

The current prompt engineering methods—such as CoT prompting,^25^ self-consistency,^26^ and DECOMP^89^—improve accuracy and reasoning quality. However, they often use the distributional cues contained within massive training corpora. Even more elaborate frameworks, such as ToT,^86^ GoT,^88^ or MaPLe,^143^ and interactive strategies, such as active prompting,^90^ offer richer scaffolding. Nevertheless, they do not definitively prove that models have transcended advanced pattern matching to acquire human-like semantic representations. The essence of meaning—i.e., how words relate to world knowledge, how concepts are interlinked, and how context shapes interpretation—are not fully understood.

Despite the rapid progress achieved, investigations of the semantic understanding capabilities of LLMs have retained significant academic and practical importance. From a scholarly perspective, true semantic grounding is a foundational challenge that spans AI, cognitive science, and linguistics.^342^ Distinguishing authentic conceptual grasp from correlation-driven mimicry requires robust evaluations. Benchmarks such as PrOntoQA^79^ test whether reasoning processes align with logical structures rather than superficial patterns. Similarly, integrating external world-knowledge embeddings and multimodal signals^16^^,^^137^^,^^343^ can help anchor the reasoning process in semantic reality, encouraging models to form stable conceptual links rather than relying on textual coincidences.

As LLMs increasingly find roles in sensitive applications—medical guidance, legal analysis, and scientific inference—distinguishing pattern-based fluency from genuine semantic understanding becomes not just an academic curiosity but a pragmatic necessity. Without rigorous inquiries into semantic grounding, even highly capable models risk producing misinterpretations or factual distortions. In other words, the better these models become at mimicking human-like output, the more pressing it is to ensure that their “understanding” is not an illusion but, rather, anchored in coherent semantic frameworks and factual correctness. Therefore, the scholarly pursuit of semantic understanding stands as a central frontier in AI research, which, in turn, will impact the future of prompt engineering research.

## Conclusion

In conclusion, prompt engineering has been established as an essential technique for optimizing the performance of LLMs. By employing foundational methods such as clear instructions and role-based prompting, in addition to advanced methodologies such as CoT and self-consistency, the performance of LLMs can be significantly enhanced. For VLMs, innovative strategies such as CoOp and MaPLe can ensure effective integration and optimization of visual and textual data. The efficiency of these methods can be rigorously assessed through subjective and objective evaluations, confirming their impacts across diverse applications, including education, content creation, programming, and complex AI systems such as agents. Moreover, prompt engineering plays an important role in the security of LLMs, which remains a critical challenge, with risks ranging from adversarial attacks to prompt manipulation. Practical solutions include prompt validation, adversarial training, and secure data governance schemes for ensuring the safe and ethical use of AI. Future prompt engineering advances could depend on attaining a deeper understanding of model structures and greater semantic understanding. This comprehensive review highlights the transformative potential of prompt engineering for advancing the capabilities of AI and providing possible directions for future research and applications.

## Acknowledgments

The authors acknowledge support from the Interdisciplinary Intelligence Super-Computer Center of Beijing Normal University at Zhuhai. This work was partially funded by the National 10.13039/501100001809Natural Science Foundation of China, China (grant no. 12271047); the Guangdong Provincial/Zhuhai Key Laboratory of Interdisciplinary Research and Application for Data Science, Beijing Normal-Hong Kong Baptist University (grant no. 2022B1212010006); BNBU research grants (grant no. UICR0400008-21, grant no. R72021114, grant no. UICR0400036-21CTL, grant no. UICR04202405-21, and grant no. UICR0700041-22); and the Guangdong College Enhancement and Innovation Program (grant no. 2021ZDZX1046).

## Author contributions

B.C. and S.Z. contributed to the initialization and conceptualization of the project. B.C., N.L., S.Z., and Z.Z. designed the overall structure of the survey. B.C. conceptualized and developed most figures, tables, and prompt examples. Z.Z. contributed to the final formatting and stylistic enhancements. B.C. drafted the basics of prompt engineering, advanced methodologies, methodologies for MMLMs, and LLM security sections. Z.Z. drafted the assessing the efficacy of prompt methods and applications improved by prompt engineering sections. B.C. and Z.Z. collaborated on the introduction and prospects sections. B.C. integrated and refined the manuscript and improved its structure. Z.Z. improved its coherence. N.L. provided strategic feedback on the overall framework, clarity, and language refinements. N.L. and S.Z. supervised and acquired funding. All authors read and approved the final manuscript.

## Declaration of interests

The authors declare no competing interests.

## Declaration of generative AI and AI-assisted technologies in the writing process

The authors acknowledge the use of generative AI tools, including ChatGPT, Claude, Perplexity, Grammarly and Writefull, in the preparation of this paper. These tools were used under the close supervision of the authors to assist in making grammar corrections, improve the logical structure and readability of the text, and gather background information. In certain sections, AI tools were used to generate initial drafts or suggestions, particularly providing background descriptions and introductions to key concepts. These outputs were thoroughly reviewed, fact-checked, and edited by the authors to ensure their scientific accuracy, clarity, and alignment with the objectives of the paper. The synthesis of the literature and the production of all figures and visual representations are the authors’ original contributions, which are on the basis of their in-depth understanding of the referenced studies. The authors affirm that the use of AI tools adheres to Elsevier’s policies on the ethical and appropriate use of generative AI in scientific writing. The authors assume full responsibility for the integrity and accuracy of all content in this document.
